# Current Usage of Extracorporeal Photopheresis in Solid Organ Transplantations in Europe: A Narrative Review

**DOI:** 10.3389/ti.2025.14906

**Published:** 2025-11-03

**Authors:** Sarah-Jane Cashmore, Markus Johannes Barten, Fritz Diekmann, Eric Epailly, Andrew J. Fisher, Andrew R. Gennery, Ben Gibbons, Johannes Gökler, Julie Guest, Anne-Elisabeth Heng, James A. Hutchinson, Teresa Rampino, Robin Vos, Luciano Potena

**Affiliations:** ^1^ Bryter Inc., New York, NY, United States; ^2^ Department of Cardiovascular Surgery, University Heart and Vascular Center Hamburg, University Medical Center Hamburg-Eppendorf, Hamburg, Germany; ^3^ Department of Nephrology and Kidney Transplantation of the Hospital Clínic, Barcelona, Spain; ^4^ Department of Cardiology and Cardiovascular Surgery, Hôpitaux Universitaires de Strasbourg, Strasbourg, France; ^5^ Translational and Clinical Research Institute, Faculty of Medical Science, Newcastle University, United Kingdom; ^6^ Department of Cardiac Surgery, Medical University of Vienna, Vienna, Austria; ^7^ Population Health Sciences Institute, Newcastle University, Newcastle, United Kingdom; ^8^ Nephrology, Dialysis Department, Clermont-Ferrand University Hospital, Clermont-Ferrand, France; ^9^ Department of Surgery, University Hospital Regensburg, Regensburg, Germany; ^10^ Nephrology, Dialysis and Transplantation Unit, Fondazione Istituto di Ricovero e Cura a Carattere Scientifico (IRCCS) Policlinico San Matteo, University of Pavia, Pavia, Italy; ^11^ Department of Respiratory Diseases and KU Leuven, Department of CHROMETA, Laboratory of Respiratory Diseases and Thoracic Surgery (BREATHE), University Hospitals Leuven, Leuven, Belgium; ^12^ Heart Failure and Transplant Unit, IRCCS Azienda Ospedaliero-Universitaria di Bologna, Bologna, Italy

**Keywords:** transplant, extracorporeal photopheresis, solid organ transplant, ECP, solid organ transplantation (SOT), SOT

## Abstract

Solid organ transplantation (SOT) faces significant challenges in managing allograft rejection, with current immunosuppressive therapies often associated with substantial adverse effects. Extracorporeal photopheresis (ECP) has emerged as a promising adjunctive treatment for rejection prevention and management in heart and lung transplants, with growing evidence supporting its use in kidney and liver transplants. Despite this, the availability of ECP and its place in standard treatment pathway is widely variable across Europe. This narrative review, supported by a European survey of 51 transplant clinicians, highlights the current usage of ECP in SOT. Findings reveal that ECP is primarily used for recurrent rejection in heart and lung transplants, with limited application currently in kidney and liver transplants. ECP has shown some efficacy in managing acute and chronic rejection, and stabilizing graft function. Barriers including lack of standardized protocols, availability of ECP, lack of high-quality clinical trial data and lack of a defined mechanism of action hinder its broader adoption. Future directions include the development of standardized protocols, multicenter registries, and further controlled clinical trials to define the role of ECP. Increased awareness, cost-effectiveness studies, mechanistic studies and equitable access are essential to integrate ECP into routine SOT management.

## Introduction

Prevention and management of allograft rejection urgently require more effective and safer therapeutic solutions. Current immunosuppressive therapies used in solid organ transplantation (SOT) are associated with substantial adverse effects, and there is a need for therapies that can provide immunomodulation while minimizing the negative impact of immunosuppression [1–4]. Extracorporeal photopheresis (ECP) is an immunomodulatory therapy currently recommended in international guidelines as an adjunctive treatment for the prevention and management of organ rejection in heart and lung transplantations, with growing evidence supporting its use in kidney and liver transplantations as well.

ECP involves the collection of leukocytes, their exposure to a photosensitizing agent (8-methoxypsoralen), ultraviolet A (UVA) light, and subsequent reinfusion into the patient [5]. This process has been shown to dampen immune responses, making it particularly valuable in the context of SOT, where balancing immune suppression to prevent rejection while minimizing infection risk is critical. Initially approved for the treatment of T-cell cutaneous lymphomas [6], ECP has since been used in graft *versus* host disease (GvHD) developed after allogeneic hematopoietic-cell transplantation and rejection of transplanted solid organs [7].

Despite the growing body of evidence supporting the use of ECP in SOT, access to ECP and its application remains inconsistent across European transplant centers [3]. Variability in clinical protocols, limited awareness among healthcare providers, lack of understanding of ECP mechanisms and the high upfront costs of ECP equipment are some of the barriers to its widespread adoption [8–10].

This narrative review is supported by a recent survey conducted by ESOT and Bryter Inc that aimed to understand current usage of ECP in solid organ transplantations in Europe. A 25-min online questionnaire was conducted May-August 2024 in accordance with privacy and data protection codes of conduct. The study complied with ethical and privacy principles for research such as the Declaration of Helsinki and GDPR. The usability and technical functionality of the electronic questionnaire had been tested before fielding the survey. The sampling frame consisted of transplant clinicians who were recruited through targeted lists provided by ESOT and were screened and profiled to ensure good representation of the European market. Transplant surgeons/cardiologists/nephrologists/pulmonologists, cardiac surgeons, general surgeons, nephrologists, and hepatologists involved in post-operative treatment and management of patients with solid organ transplantation (kidney, heart, lung, or liver) were invited to take part. Informed consent was obtained from participants at the beginning of the survey, and no personal or identifiable data was collected. Out of a total of n = 734, n = 51 completed the survey. The sample consisted of n = 51 clinicians across Europe: Belgium, Denmark, Finland, France, Germany, Greece, Italy, Netherlands, Norway, Romania, Spain, Switzerland, and the United Kingdom.

## Current Usage of ECP in Solid Organ Transplantation

### European Survey Findings

**FIGURE 1:**
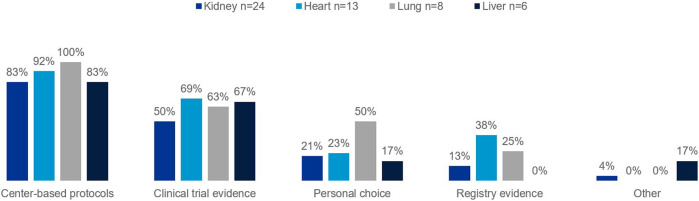
Percentage of European clinicians using each of the following as part of their clinical rationale for approach taken regarding treatments to prevent against rejection in transplant recipients, by organ type.

The European survey found center-based protocols are the primary clinical rationale for the approach taken regarding treatments to prevent post-transplant rejection across organ types (Figure 1). Clinical trial evidence informs protocols, and the majority of clinicians will take trial evidence into consideration when deciding on treatments.

**FIGURE 2:**
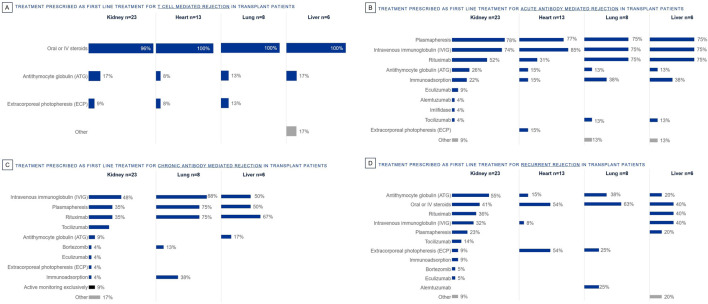
Percentage of European clinicians using each treatment first line by organ type, for the following rejection types: **(A)** T Cell Mediated Rejection, **(B)** Acute Antibody Mediated Rejection, **(C)** Chronic Antibody Mediated Rejection, **(D)** Recurrent Rejection.

Oral or IV steroids are the most prescribed first line treatment for T cell mediated rejection in solid organ transplant (Figure 2A). For acute antibody mediated rejection, plasmapheresis, IVIG and Rituximab are the most prescribed first line treatments (Figure 2B). Likewise for chronic antibody mediated rejection, IVIG, plasmapheresis and Rituximab are the most prescribed first line treatments (Figure 2C). ATG, Oral or IV steroids, Rituximab, and IVIG are the most prescribed first line treatments for recurrent rejection (Figure 2D). First line treatments prescribed are determined by rejection type; ECP is used only by some in first line, specifically in T cell mediated (lung 13%, kidney 9%, heart 8%), acute antibody mediated (heart 15%) or recurrent (heart 54%, lung 25%, kidney 9%) rejection.

**FIGURE 3:**
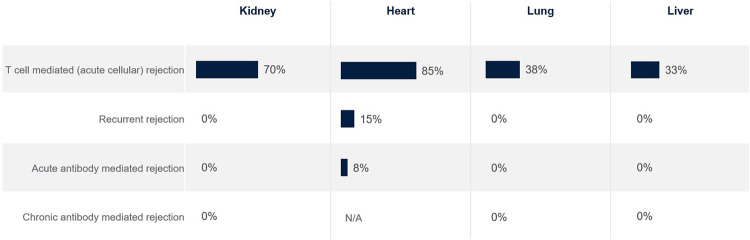
Percentage of European clinicians ‘very satisfied’ with current treatments for transplant recipients, by organ type.

Satisfaction with current treatments is very low for almost all rejection types, except for T cell mediated rejection in kidney and heart transplantation (but not lung and liver) (Figure 3). T cell mediated rejection often responds well to standard treatments, protocols are well-established and have clear diagnostic criteria. There is a major need for more efficacious and well tolerated treatments across all rejection types.

**FIGURE 4:**
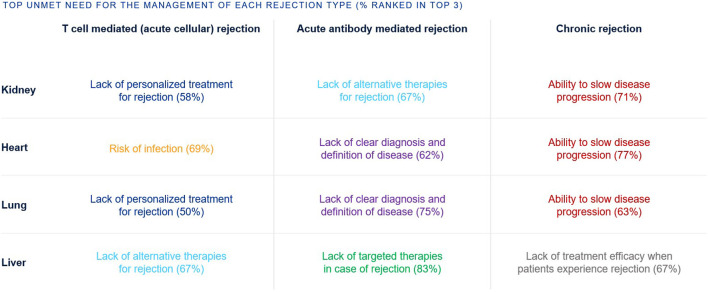
Percentage of European clinicians ranking each unmet need in the top 3 unmet needs for the management of transplant recipients, by rejection type and organ type.

The top unmet needs in SOT tend to be grouped per rejection type rather than organ type (Figure 4). For T cell mediated rejection, top unmet needs were a lack of personalized treatment for rejection (kidney, lung), as well as risk of infection (heart) and lack of alternative therapies (liver). For acute antibody mediated rejection, top unmet needs were lack of clear diagnosis and disease definition (heart, lung), lack of alternative therapies (kidney), and lack of targeted therapies (liver). For chronic rejection, top unmet needs were ability to slow disease progression (kidney, heart, lung) and lack of treatment efficacy (liver).

**FIGURE 5:**
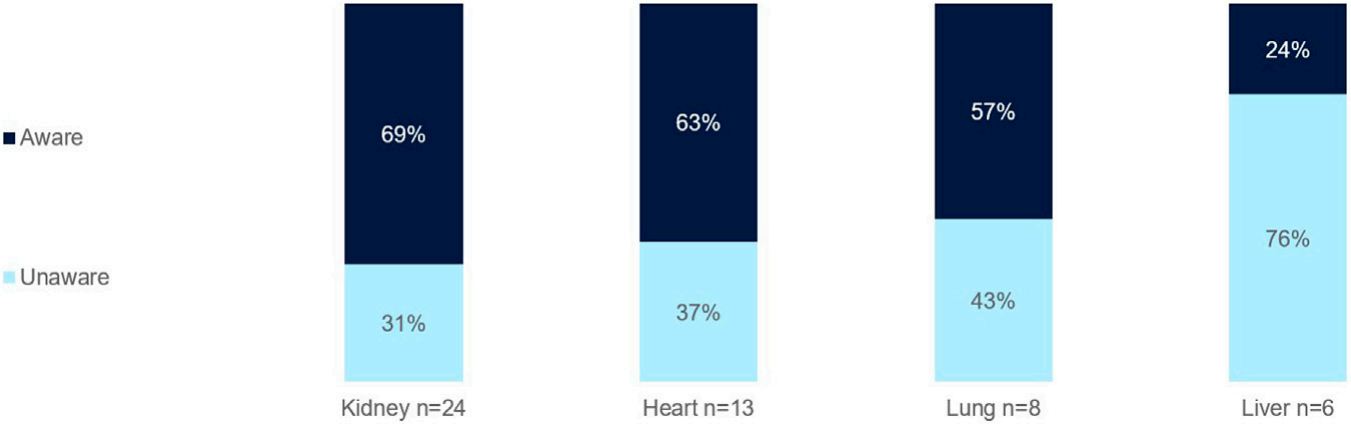
Percentage of European clinicians aware of ECP as a treatment option for SOT per organ type.

Most clinicians surveyed were aware of ECP as a therapy option for kidney (69%), heart (63%), and lung (57%) transplants (Figure 5). Fewer were aware of ECP for liver transplants (24%).

**FIGURE 6:**
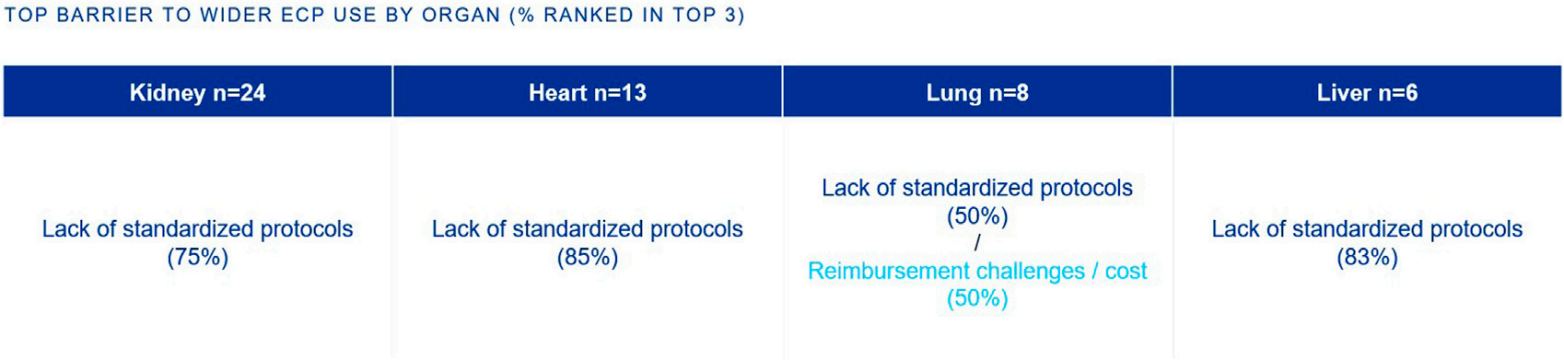
Percentage of European clinicians ranking each barrier in the top 3 barriers to wider ECP use, by organ type.

The top barriers to the widespread adoption of ECP in SOT is the lack of standardized clinical protocols (kidney, heart, lung, liver) and reimbursement/cost challenges (lung) (Figure 6).

**FIGURE 7:**
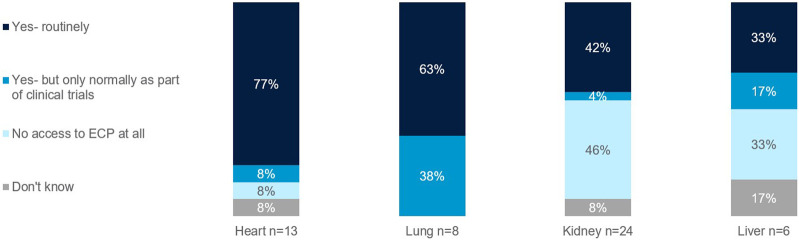
Rates of routine access to ECP. Percentage of European clinicians treating transplants of the heart, lung, kidney, and liver.

**FIGURE 8:**
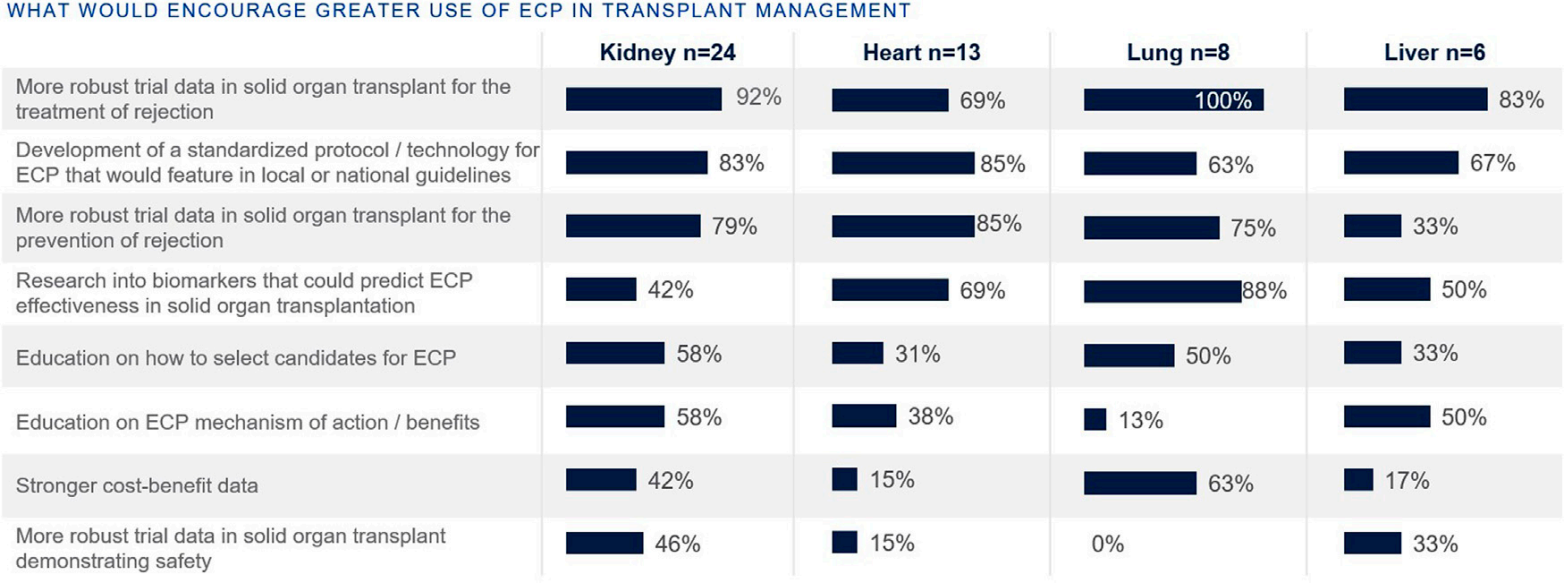
Percentage of European clinicians agreeing each factor would encourage greater ECP use, by organ type.

Those focused on heart and lung transplants have higher rates of routine access to ECP as a therapy option at their centers (77% and 63%, respectively), whereas the rate is lower for those focused on kidney and liver (42% and 33%, respectively) (Figure 7). Those with no access to ECP at all were those who treat kidney (46%), liver (33%), and heart (8%). When asked what would encourage use of ECP in transplant management, top reasons included more robust clinical trial data and the development of a standardized protocol (Figure 8).

### ECP in Solid Organ Transplantation

#### Heart Transplantation

A major cause of mortality and morbidity in heart transplant recipients is cardiac allograft vasculopathy (CAV) [11]. Acute rejection is also a significant risk in the first year following heart transplantation [12]. ECP has been widely studied in heart transplantation, particularly for acute cellular rejection (ACR) and antibody-mediated rejection (AMR). It is useful in cases where patients suffer from steroid-resistant or recurrent rejection or where reducing immunosuppressive drug toxicity is a priority. ECP has been employed as an adjunct therapy to dampen immune responses and decrease dosage of standard immunosuppressive regimens, which are associated with events such as renal insufficiency, increased infection, and neurotoxicity [11, 13].

In heart transplantation, acute antibody-mediated rejection is a significant cause of early graft dysfunction. ECP has been used as a rescue therapy in refractory AMR, with case reports and small studies suggesting improved hemodynamic stability and graft function [3]. The exact mechanism remains unclear but is likely related to the modulation of alloimmune responses and the lack of power in available studies limits the ability to draw strong conclusions. The American Heart Association also noted there are no published data conclusively demonstrating efficacy of ECP in AMR in SOT, and mechanistic understanding remains incomplete [14]. It notes that while ECP has been successfully used for recurrent rejection and acute cellular rejection, its role in AMR remains undefined and warrants further investigation. ECP is not included as a standard or first-line therapy for AMR in the American Heart Association’s treatment algorithms, and only considered in refractory cases [14]. ECP is generally well received in steroid-refractory ACR in heart transplantation. Preliminary data in small sample sizes found ECP to be well tolerated and reduce the dose of standard of care immunosuppression, as well as a reduction in the number of rejection episodes [15, 16]. A retrospective chart review of 102 heart transplant patients treating rejection with ECP found 88.2% of patients remained rejection-free despite lower dose in standard of care immunosuppression and 92.3% had reduced rejection grades [17]. However, as well as limitations associated with the retrospective design, the effectiveness of ECP in comparison with other treatment options was not assessed due to the descriptive, single-arm design. A single center retrospective study on 22 patients assessed standard of care immunosuppression plus ECP, and found zero episodes of ISHLT grade 3R ACR and two episodes of 2R ACR episodes were reversed over the study period [18]. Furthermore, decreased rates of subsequential rejection episodes, and normalized allograft function were observed in patients completing the ECP course [18]. However, these findings were from a small sample in a single center, and large scale randomized clinical trials are required to validate this.

In heart transplantation, chronic rejection manifests as cardiac allograft vasculopathy. Studies have demonstrated that ECP can slow the progression of CAV by reducing immune-mediated endothelial injury [14, 17, 19, 20].

Savignano et al [11] performed a retrospective case series on 8 patients treated with ECP for recurrent rejection. Of the 8, 3 patients had negative biopsies with no rejection at the end of treatment; 4 patients showed no response to ECP; 1 could not be evaluated [11]. A single-center study reported on 20 pediatric heart transplant patients who underwent ECP, showing that it can be safely applied in this population [21]. The study found a survival rate of 84% at 1 year and 53% at 3 years post-ECP initiation.

#### Lung Transplantation

Lung transplantation is associated with a high risk of chronic lung allograft dysfunction (CLAD), most commonly bronchiolitis obliterans syndrome (BOS) [13]. Current immunosuppression strategies and its modifications do not reverse BOS but instead aim to stabilize or slow its progression [13, 22]. In lung transplantation, although some studies suggest ECP benefit in reducing acute cellular rejection and CLAD, earlier reviews highlight that no randomized controlled trials have yet confirmed these findings, and most available data are from small, single-center, non-randomized studies, limiting generalizability and strength of evidence [23]. [24] There are currently no guidelines on early prophylactic ECP in lung transplantation, but this is presently being evaluated [25].

A recent randomized controlled trial evaluated the prophylactic addition of ECP to standard immunosuppressive therapy in lung transplant recipients, and demonstrated a significant reduction in ACR episodes, lymphocytic bronchiolitis, and CLAD within 24 months [26]. In this study, the ECP group also experienced fewer severe infections and adverse events compared to the control group [26]. This study was the first prospective RTC in lung transplant recipients evaluating early use of ECP in addition to standard triple immunosuppressive therapy. Although an RTC provides stronger evidence, this study had a small sample size (n = 31 per group) and only patients transplanted for COPD were included.

Further evidence is needed to confirm the impact of ECP in lung transplant AMR. One analysis has found that ECP is able to reduce circulating *de novo* donor-specific antibodies (dnDSA) (cleared in 88% of patients), and lung function was restored in 38% patients [27]. This was however a single-center retrospective analysis, and as such limits generalizability and lacks causality.

Chronic lung allograft dysfunction, including bronchiolitis obliterans syndrome, is a leading cause of late graft failure in lung transplantation. ECP has been investigated as a therapeutic option for CLAD/BOS [24]. ECP has been used as a second-line therapy for BOS, with studies reporting stabilization or improvement in lung function in some patients [28–32]. Stronger evidence comes from Benazzo et al [33] that assessed ECP use in 631 patients (87% BOS, 13% RAS) from 3 European centers and found long-term stabilization of lung function was achieved in 42% of patients with improvement in 9% [33]. This study was not free of limitations however; it was retrospective (indications of ECP may change over time and possibilities of miscoding data), data on AMR and DSAs were not included, and clinical practice might differ between the centers. Another retrospective study on 373 CLAD patients were initiated onto ECP after ≥10% decline in FEV1 from baseline. Statistical modeling revealed 5 different temporal CLAD phenotypes based on the FEV1 course and suggested predicting survival at ECP initiation appears feasible [34]. Again, there are limitations with the retrospective study nature. Jaksch et al. [13] demonstrated that ECP could stabilize or improve lung function in some patients with BOS, however this was a single center study on n = 51 patients and lacks generalizability [13]. Another retrospective study on early ECP in CLAD (n = 105) by Gautschi et al. (2024) recommended early initiation of ECP to slow lung function decline and improve survival rates [35].

#### Liver Transplantation

The benefits of liver transplantation vary by patient, with the potential for chronic rejection or late graft loss due to disease recurrence [36]. Most published experience with ECP in liver transplantation is limited to small case series, pilot studies, and anecdotal reports, with as of yet no randomized controlled trials or large prospective studies validating its effectiveness for prevention or treatment of rejection, or for improving long-term graft or patient survival [3, 8, 37].

ECP has been explored as an adjunct to delay calcineurin inhibitor introduction, prophylaxis of acute cellular rejection in high-risk or ABO-incompatible recipients, and as a strategy to reduce immunosuppressive burden in hepatitis C virus-positive patients. However, these applications remain investigational, and the reported benefits are preliminary, with outcomes such as rejection rates and virological response requiring confirmation in larger, randomized controlled trials [38]. Further studies found preliminary data that supported the finding that ECP potentially provides a low complication rate immunomodulation in liver transplantation [37, 39]. In rare complications such as graft-versus-host disease post-liver transplant, ECP has been used in individual cases, but the evidence again is limited to case reports and does not establish efficacy or impact on survival [40, 41]. Its role remains investigational, with limited data available.

#### Kidney Transplantation

There is a lack of clinical trials relating to ECP in kidney transplantation, with most of the current evidence for it use relating to case studies [13]. ECP has been explored as an alternative for T cell-mediated rejection when standard therapies such as corticosteroids and anti-thymocyte globulin are contraindicated or not tolerated, but its use is based on limited case series and small studies rather than randomized controlled trials [3, 8, 42]. Preliminary studies have shown that ECP can modulate cellular immunity in the long term and reduce acute glomerular lesions without causing major chronic lesions. Faenko et al. [43] suggests ECP contributes to activation of tolerogenic T-regulatory cells, maintaining long-term graft survival [43].

ECP has been used in combination with plasmapheresis, intravenous immunoglobulin (IVIG), and rituximab to treat refractory AMR in kidney transplant recipients. The immunomodulatory effects of ECP, including the induction of Tregs and suppression of B-cell activity, are thought to contribute to its efficacy. ECP was shown in one small study (n = 14) to stabilize the renal function in more than 70% of cases and significantly lower DSA levels [44].

In kidney transplantation, chronic rejection is less commonly treated with ECP compared to other organs. However, emerging evidence suggests that ECP may have a role in managing chronic antibody-mediated rejection (cAMR). A prospective observational study found that 72.7% of patients responded to ECP with stabilization of renal function for up to 3 years and even an improvement in GFR in seven cases of chronic rejection and any adverse reaction [44]. However, the overall clinical impact of ECP in kidney transplantation remains uncertain due to the lack of high-quality, large-scale studies.

#### Clinical Guidelines and Recommendations

Current clinical guidelines and consensus statements from major transplant societies and organizations, including the American Society for Apheresis and the American Society of Transplantation, recognize extracorporeal photopheresis as an adjunctive therapy for the prevention and management of rejection in heart and lung transplantation [8, 20].

In Europe, ECP is increasingly recognized as an adjunctive therapy for some patients at risk of rejection or experiencing allograft dysfunction after solid organ transplantation. The British Photodermatology Group (BPG) and the UK Cutaneous Lymphoma Group (UKCLG; formerly the UK Skin Group) support the use of ECP as a treatment for cardiac allograft rejection and rejection prophylaxis [7, 12, 45]. ECP is most commonly indicated for heart transplant patients with recurrent/refractory acute cellular rejection, those with intolerance or contraindications to standard immunosuppression, and for immunosuppression minimization [3, 8, 17, 18]. There is no universally accepted dosing protocol, and ECP is usually an adjunct to standard immunosuppressive regimens, but typical regimens involve 2-3 treatments per week for several weeks, then tapering based on response [18, 20].

The International Society for Heart and Lung Transplantation (ISHLT) also identifies ECP as an option for the treatment of chronic or resistant acute cellular rejection and for managing CLAD/BOS, especially in patients with recurrent rejection or intolerance to standard immunosuppression [25, 46, 47]. As of yet, ECP is not included in any major international, European, or United States liver or kidney transplant guidelines as a recommended therapy for rejection or immunosuppression minimization [3, 8], however ongoing research and clinical trials are exploring its role in managing rejection and reducing immunosuppressive drug toxicity in these settings.

#### ECP Efficacy and Outcomes Summary

Current standard of care immunosuppressive therapy in solid organ transplantation lacks in efficacy and has a cumulative side effect profile [48]. Standard of care immunosuppressive therapies can result in side effects such as infection, malignancy, cardiovascular diseases, and nephrotoxicity [3]. ECP is an immunomodulatory approach that provides a potential solution in both rejection treatment and rejection prophylaxis [3]. Currently however, the majority of data stems from single-center or case studies, and large-scale clinical trials are required to fully understand its potential. The effectiveness of ECP varies among different types of solid organ transplants, with the most evidence and guideline support seen in heart and lung transplants.

In heart transplantation, ECP is linked to high rates of rejection-free outcomes (up to 88%–83% in prevention and treatment groups), improvements in rejection grades on histology, and the safe reduction of immunosuppressive medications, particularly calcineurin inhibitors [17]. A small (n = 15) single center study found a significant proportion of patients (up to 64%) experience a reduction in donor-specific antibodies, along with decreases in gene expression profiling and donor-derived cell-free DNA [49]. However, survival rates are similar to registry data and do not surpass those achieved with standard therapy.

ACR and CLAD contribute to lung transplants having the worst long-term outcomes of all solid organ transplants [26]. Freedom from rejection and freedom from CLAD are significantly improved when ECP is added to standard immunosuppression [26]. It was also shown in a retrospective cohort study to slow the decline in forced expiratory volume in one second (FEV1), with a 63% reduction in the rate of FEV1 decline in cases of chronic rejection [50]. Other studies (including a randomized controlled trial in lung transplantation focused on ECP for prevention of rejection and chronic lung allograft dysfunction) reported stabilization or improvement in FEV1 [22, 26, 51]. Additionally, ECP promotes the reduction and clearance of donor-specific antibodies and antibodies targeting lung-specific antigens, which is associated with improved lung function and decreased levels of pro-inflammatory cytokines [3, 8, 23].

ECP provides measurable improvements in freedom from rejection, FEV1, and DSA reduction in heart and lung transplantation, but data are insufficient to support similar benefits in liver and kidney transplantation [3, 49]. In kidney and liver transplantation, ECP is considered investigational or reserved for refractory cases. There is insufficient evidence for reduction in ACR, improvement in DSA kinetics, or preservation of renal function, and ECP is not guideline-recommended for routine use in these settings [3, 17].

## Challenges and Unmet Needs

### Unmet Clinical Needs in SOT

The challenges of SOT are multifactorial, with different rejection types having a different set of unmet needs (Figure 4). The top unmet needs tend to be grouped by rejection type rather than organ type.

Although T-cell mediated/acute cellular rejection often responds well to increased immunosuppressive therapy, a minority of patients can be refractory and warrant the need for alternative therapies for rejection. Due to the nature of this rejection type, there is also a need for personalized treatments.

In antibody mediated rejection (AMBR) treatment resistance can also be observed, warranting the need for alternative therapies [52]. In SOT there is a risk of developing acute AMBR in patients who develop *de novo* DSA following transplantation or in patients who have pre-formed DSA to HLA at the point of transplantation [52]. These patients who have developed acute AMBR may progress to chronic rejection and are highly associated with graft loss [52]. However, due to the complexity of its pathology and unclear cellular/molecular pathways, acute AMBR lacks a clear definition of the disease and therefore a clear diagnosis [52–54].

Chronic rejection is characterized by repeated injury of the graft vasculature and uncontrolled repair responses that can result in transplant vasculopathy [14, 52, 55]. Chronic rejection resulting in transplant vasculopathy is leading cause of re-transplantation with a significant impact on patient mortality and morbidity [56, 57]. Currently chronic rejection is irreversible, and a major unmet need is the ability to slow disease progression.

### Limitations of Current Mechanistic Research on ECP in Transplantation

One of the most critical gaps in current knowledge is the incomplete understanding of the mechanism of action of ECP. Several mechanisms have been hypothesized as to how ECP modulates the immune system: early theories suggest apoptosis of treated leukocytes due to the combination of psoralen and ultraviolet A [58, 59], and later theories suggest transimmunization via differentiation of immature dendritic cells [60–62], modification of the cytokine profile [63–65], and stimulation of several T-cell lineages (in particular regulatory T-cells) [66, 67]. However, the precise pathways and cellular interactions remain unclear. This lack of mechanistic clarity hinders the ability to refine ECP protocols for specific transplant-related conditions and patient populations [8, 68]. There is increasing research attempting to elucidate the mechanism of action in order to extend the use of ECP and better target its use in current indications [61, 69].

Currently, ECP demonstrates organ-specific benefits, with the most evidence for DSA reduction and stabilization in lung transplantation, while results in heart, kidney, and liver transplantation are more equivocal/insufficiently established [3, 8, 17, 22, 23, 26, 49, 50]. DSAs are a critical marker of antibody-mediated rejection and graft survival in SOT. If ECP shows inconsistent or unclear results in reducing DSAs, clinicians may hesitate to recommend it as a reliable therapy, especially when other treatments (e.g., plasmapheresis, IVIG, or rituximab) have more established efficacy in DSA management.

### Challenges in Implementing ECP in Clinical Practice Across Europe

Awareness of ECP as a treatment option in SOT is the first barrier to its use. Fewer clinicians surveyed were aware of ECP as a therapy option for liver transplants, as opposed to the higher awareness for heart, lung, and kidney transplants (Figure 5). This is likely due to the limited ECP research in the area and the fact that liver transplants are generally more immunologically tolerant compared to heart or kidney transplants, which may reduce the perceived need for adjunctive therapies like ECP. Acute and chronic rejection rates in liver transplantation are lower, and standard immunosuppressive regimens are often sufficient.

A top barrier to the widespread adoption of ECP in SOT is the lack of standardized clinical protocols (Figure 6). While ECP is recommended in international guidelines for specific indications there is significant variability in its application across transplant centers [3].

Access to ECP remains limited in many regions, including Europe. Access to ECP is unevenly distributed across Europe, with significant disparities between countries and even within regions of the same country. ECP requires specialized facilities and equipment, which are not widely available in all hospitals or regions. Larger transplant centers in urban areas are more likely to have the resources and infrastructure to offer ECP, while smaller or rural centers often lack access to this therapy. This geographic disparity creates inequities in patient care, as transplant recipients in underserved areas may not have the opportunity to benefit from ECP. Patients may need to travel long distances to access treatment, creating logistical and financial burdens. Furthermore, ECP sessions are time-consuming, often requiring several hours per session, with treatments typically repeated multiple times per week or month. This can be inconvenient for patients and resource-intensive for healthcare providers.

There are also access limits per organ type. The European survey found that those focused on heart and lung transplants have higher rates of routine access to ECP as a therapy option at their centers, whereas the rate is lower for those focused on kidney and liver (Figure 7).

A further barrier to ECP access is the high upfront cost of the equipment and treatment sessions. ECP requires specialized apheresis machines, trained personnel, and infrastructure, which can be prohibitively expensive for smaller or resource-constrained transplant centers. The medical literature acknowledges ECP’s potential to reduce immunosuppression-related adverse effects and improve clinical outcomes in heart and lung transplantation, but explicitly notes that cost-effectiveness analyses are lacking and represent a major gap in current research [3, 8]. Additionally, reimbursement policies for ECP vary widely across countries and healthcare systems, with some systems failing to cover the full cost of treatment. This financial burden limits the availability of ECP, particularly in public healthcare systems or in regions with limited healthcare funding [1, 8]. The absence of formal cost-effectiveness studies means that the economic impact of ECP remains undetermined in all solid organ transplant settings [3, 8].

## Future Directions

As research continues to demonstrate its efficacy in reducing acute and chronic rejection in heart, lung, kidney, and liver transplants, ECP is expected to become a more integral part of SOT protocols, especially for patients who are unable to tolerate standard immunosuppressive regimens [3, 8]. 59% of European clinicians surveyed agreed with the statement: “*It would be of benefit to my clinical practice if more ECP was used to treat transplant rejection in the future*”.

More robust clinical trial data and the development of a standardized protocol would encourage use of ECP in transplant management (Figure 8). Randomized clinical trial data would provide more evidence for the clinical indication and efficacy in ECP, particularly of use to kidney and liver transplants where the use of ECP currently is less established. Indeed, ECP is being actively studied across a range of clinical settings, including solid organ transplantation, chronic rejection, GvHD, and autoimmune diseases. The ongoing phase II randomized control trial E-CLAD UK assesses ECP in CLAD, and will further collect long-term follow up data [70]. There are several more actively recruiting trials, including the EUROEXPORT-DSA trial from Medical University of Vienna that will assess ECP in subclinical antibody-mediated rejection after lung transplantation (NCT06112951), a study on the impact of ECP for the prevention of acute rejection in highly sensitized kidney transplant recipients from Fundacion Clinic per a la Recerca Biomédica (NCT04414735), and the phase IIb study by University of Miami on axatilimab in combination with ECP in chronic graft-versus-host disease (NCT06663722).

Furthermore, the development of standardized ECP protocols that are incorporated into national guidelines would promote the broader adoption of ECP by providing clear, evidence-based recommendations for its use in clinical practice. Currently, the variability in ECP protocols, including differences in treatment frequency, duration, and patient selection criteria, limits its implementation and creates uncertainty among clinicians. Furthermore, standardized protocols would facilitate more consistent data collection across institutions, enabling better evaluation of clinical outcomes and cost-effectiveness.

Establishment of multicenter registries to collect real-world data on the use of ECP in SOT is essential for evaluating its long-term efficacy and safety. Such registries would provide valuable insights into patient selection criteria, treatment protocols, and outcomes, thereby informing future policy and practice improvements [9].

Further awareness and expertise among healthcare providers regarding the benefits and mechanisms of ECP can encourage its adoption [71]. Future initiatives should include targeted educational programs and training workshops for transplant clinicians, nurses, and allied healthcare professionals.

Future research should focus on exploring the role of ECP in pediatric SOT recipients. While ECP has shown some promise in pediatric heart transplantation [21], data on its use in solid organ transplants in pediatric patients are limited. Given its use and efficacy in managing acute and chronic GvHD in pediatric patients following bone marrow transplantation [72–74], investigating its potential benefits in SOT could help establish evidence-based guidelines for its use in this patient population.

The successful integration of ECP into SOT management requires collaboration among transplant specialists, immunologists, policymakers, and industry stakeholders. Interdisciplinary efforts should focus on addressing logistical challenges, streamlining treatment pathways, and ensuring equitable access to ECP across diverse healthcare settings [4].

Cost-effectiveness studies have shown that ECP can reduce long-term healthcare costs by decreasing the need for high-dose immunosuppressive therapies and reducing the incidence of graft loss and associated complications [3]. However, these benefits are often not immediately apparent, leading to underinvestment in ECP programs.

## Limitations

This review has a number of limitations that will now be discussed. The survey data used to support this review whilst targeted to be broad, may not be fully generalizable. The sample size was relatively small (n = 51) and limited to clinicians from specific European countries. This regional focus may not reflect the practices, challenges, or perspectives of transplant centers in other regions of the world. While the survey provides valuable insights into current practices and perceptions, it relies on self-reported data, which by its nature may be subject to recall bias or variability in interpretation. The design of a narrative review inherently does not have a full formal systematic search and exclusion strategy. Finally, significant evidence gaps remain regarding the efficacy, mechanisms of action, and optimal protocols for ECP in solid organ transplantation, particularly for kidney and liver transplants. These gaps highlight the need for further large-scale, multicenter randomized controlled trials to better understand the role of ECP in this context.

## Conclusion

The approach to treatment in solid organ transplantation is primarily guided by established center protocols, evidence from experience, and to a lesser extent clinical trials. However, there is widespread dissatisfaction with current treatments across various organs and types of rejection. First-line treatments are selected based on the type of rejection, with extracorporeal photopheresis being used by some as a first-line option, especially for recurrent rejection in heart and lung transplants. There are significant unmet needs in managing solid organ transplant patients, particularly in treating rejection, managing immunosuppression, and improving diagnostics.

ECP is recognized as an effective adjunctive therapy for managing organ rejection in some recipients, particularly in heart and lung transplantations. ECP offers immunomodulatory benefits that can help reduce the need for traditional immunosuppressive therapies, which are often associated with significant side effects such as infections, malignancies, and nephrotoxicity. Awareness and access to ECP vary by organ, and its usage is currently low, unaided by mechanistic uncertainty. The main barrier to wider ECP use is the absence of standardized protocols across different organs. Despite a generally positive perception of ECP’s benefits in treating transplant rejection, there is a lack of awareness regarding its efficacy. More clinicians have routine access to ECP for heart and lung transplants than for kidney and liver. The development of standardized protocols could encourage greater use of ECP. While current evidence supports its use, there is a need for further research, including randomized controlled trials, to better understand its full potential and optimize its use across different types of solid organ transplants.

## References

[1] Ruiz R Kirk AD. Long-Term Toxicity of Immunosuppressive Therapy. In: Transplantation Of the Liver 1354–1363. Elsevier (2015). 10.1016/B978-1-4557-0268-8.00097-X

[2] Szumilas K Wilk A Wiśniewski P Gimpel A Dziedziejko V Kipp M Current Status Regarding Immunosuppressive Treatment in Patients after Renal Transplantation. Int J Mol Sci (2023) 24:10301. 10.3390/ijms241210301 37373448 PMC10298917

[3] Augusto J-F Benden C Diekmann F Zuckermann A. The Value of Extracorporeal Photopheresis as an Immunosuppression-Modifying Approach in Solid Organ Transplantation: A Potential Solution to an Unmet Medical Need. Front Immunol (2024) 15:1371554. 10.3389/fimmu.2024.1371554 38846942 PMC11154098

[4] Pilch NA Bowman LJ Taber DJ. Immunosuppression Trends in Solid Organ Transplantation: The Future of Individualization, Monitoring, and Management. Pharmacother The J Hum Pharmacol Drug Ther (2021) 41:119–31. 10.1002/phar.2481 33131123 PMC8778961

[5] Parrish JA Fitzpatrick TB Tanenbaum L Pathak MA. Photochemotherapy of Psoriasis with Oral Methoxsalen and Longwave Ultraviolet Light. New Engl J Med (1974) 291:1207–11. 10.1056/NEJM197412052912301 4422691

[6] Edelson R Berger C Gasparro F Jegasothy B Heald P Wintroub B Treatment of Cutaneous T-Cell Lymphoma by Extracorporeal Photochemotherapy. Preliminary Results. New Engl J Med (1987) 316:297–303. 10.1056/NEJM198702053160603 3543674

[7] Knobler R Berlin G Calzavara-Pinton P Greinix H Jaksch P Laroche L Guidelines on the Use of Extracorporeal Photopheresis. J Eur Acad Dermatol Venereol (2014) 28:1–37. 10.1111/jdv.12311 24354653 PMC4291097

[8] Barten MJ Fisher AJ Hertig A. The Use of Extracorporeal Photopheresis in Solid Organ Transplantation—Current Status and Future Directions. Am J Transplant (2024) 24:1731–41. 10.1016/j.ajt.2024.03.012 38490642

[9] European Society for Organ Transplantation (ESOT). Repository for Extracorporeal Photopheresis Evidence (ESOT-RECPE). Repository for ECP Evidence (2024).

[10] exTra Doctoral Network. Innovative Applications of Extracorporeal Photopheresis in Transplantation (2024). Available online at: https://extra-horizon.eu/ (Accessed March 27, 2025).

[11] Savignano C Rinaldi C Tursi V Dolfini C Isola M Livi U Extracorporeal Photochemotherapy in Heart Transplant Rejection: A Single-Center Experience. Transfus Apher Sci (2017) 56:520–4. 10.1016/j.transci.2017.07.009 28774825

[12] McKenna KE Whittaker S Rhodes LE Taylor P Lloyd J Ibbotson S Evidence-based Practice of Photopheresis 1987-2001: A Report of a Workshop of the British Photodermatology Group and the U.K. Skin Lymphoma Group. Br J Dermatol (2006) 154:7–20. 10.1111/j.1365-2133.2005.06857.x 16403088

[13] Jaksch P Knobler R. ECP and Solid Organ Transplantation. Transfus Apher Sci (2014) 50:358–62. 10.1016/j.transci.2014.04.006 24768429

[14] Colvin MM Cook JL Chang P Francis G Hsu DT Kiernan MS Antibody-mediated Rejection in Cardiac Transplantation: Emerging Knowledge in Diagnosis and Management: A Scientific Statement from the American Heart Association. Circulation (2015) 131:1608–39. 10.1161/CIR.0000000000000093 25838326

[15] DallʼAmico R Livi U Milano A Montini G Andreetta B Murer L Extracorporeal Photochemotherapy as Adjuvant Treatment of Heart Transplant Recipients with Recurrent Rejection. Transplantation (1995) 60:45–9. 10.1097/00007890-199507150-00009 7624941

[16] Maccherini M Diciolla F Laghi Pasini F Lisi G Tanganelli P D'Ascenzo G Photopheresis Immunomodulation after Heart Transplantation. Transpl Proc (2001) 33:1591–4. 10.1016/s0041-1345(00)02605-1 11267432

[17] Barten MJ Sax B Schopka S Amarelli C Epailly E Natali B European Multicenter Study on the Real-World Use and Clinical Impact of Extracorporeal Photopheresis after Heart Transplantation. The J Heart Lung Transplant (2023) 42:1131–9. 10.1016/j.healun.2023.03.005 37037751

[18] Teszak T Assabiny A Kiraly A Tarjanyi Z Parazs N Szakal-Toth Z Extracorporeal Photopheresis in the Treatment of Cardiac Allograft Rejection: A Single-Centre Experience. Transpl Immunol (2023) 79:101853. 10.1016/j.trim.2023.101853 37196865

[19] Barr ML Baker CJ Schenkel FA McLaughlin SN Stouch BC Starnes VA Prophylactic Photopheresis and Chronic Rejection: Effects on Graft Intimal Hyperplasia in Cardiac Transplantation. Clin Transpl (2000) 14:162–6. 10.1034/j.1399-0012.2000.140211.x 10770423

[20] Slomovich S Bell J Clerkin KJ Habal MV Griffin JM Raikhelkar JK Extracorporeal Photopheresis and its Role in Heart Transplant Rejection: Prophylaxis and Treatment. Clin Transpl (2021) 35:e14333. 10.1111/ctr.14333 33914369

[21] Carlo WF Pearce FB George JF Tallaj JA McGiffin DC Marques MB Single-Center Experience With Extracorporeal Photopheresis in Pediatric Heart Transplantation. The J Heart Lung Transplant (2014) 33:624–8. 10.1016/j.healun.2014.01.863 24661684

[22] Benden C Speich R Hofbauer GF Irani S Eich-Wanger C Russi EW Extracorporeal Photopheresis after Lung Transplantation: A 10-Year Single-Center Experience. Transplantation (2008) 86:1625–7. 10.1097/TP.0b013e31818bc024 19077900

[23] Hachem R Corris P. Extracorporeal Photopheresis for Bronchiolitis Obliterans Syndrome after Lung Transplantation. Transplantation (2018) 102:1059–65. 10.1097/TP.0000000000002168 29557913 PMC7228625

[24] Jaksch P Scheed A Keplinger M Ernst MB Dani T Just U A Prospective Interventional Study on the Use of Extracorporeal Photopheresis in Patients with Bronchiolitis Obliterans Syndrome after Lung Transplantation. The J Heart Lung Transplant (2012) 31:950–7. 10.1016/j.healun.2012.05.002 22884382

[25] Knobler R Arenberger P Arun A Assaf C Bagot M Berlin G European Dermatology Forum: Updated Guidelines on the Use of Extracorporeal Photopheresis 2020 – Part 2. J Eur Acad Dermatol Venereol (2021) 35:27–49. 10.1111/jdv.16889 32964529 PMC7821314

[26] Benazzo A Cho A Auner S Schwarz S Kovacs Z Ramazanova D Extracorporeal Photopheresis for the Prevention of Rejection After Lung Transplantation: A Prospective Randomised Controlled Trial. Eur Respir J (2025) 65:2400733. 10.1183/13993003.00733-2024 39638420 PMC11799887

[27] Benazzo A Worel N Schwarz S Just U Nechay A Lambers C Outcome of Extracorporeal Photopheresis as an Add-On Therapy for Antibody-Mediated Rejection in Lung Transplant Recipients. Transfus Med Hemother (2020) 47:205–13. 10.1159/000508170 32595425 PMC7315205

[28] Leroux J Hirschi S Essaydi A Bohbot A Degot T Schuller A Initiation of Extracorporeal Photopheresis in Lung Transplant Patients with Mild to Moderate Refractory BOS: A Single-Center Real-Life Experience. Respir Med Res (2022) 81:100913. 10.1016/j.resmer.2022.100913 35525096

[29] Karnes HE Schindler EI Morrell M Hachem RR Berman K Vedantham S Factors Associated with Mortality and Response to Extracorporeal Photopheresis in Lung Allograft Recipients with Bronchiolitis Obliterans Syndrome. Transplantation (2019) 103:1036–42. 10.1097/TP.0000000000002430 30130331

[30] Moniodis A Townsend K Rabin A Aloum O Stempel J Burkett P Comparison of Extracorporeal Photopheresis and Alemtuzumab for the Treatment of Chronic Lung Allograft Dysfunction. The J Heart Lung Transplant (2018) 37:340–8. 10.1016/j.healun.2017.03.017 28431983

[31] Pecoraro Y Carillo C Diso D Mantovani S Cimino G De Giacomo T Efficacy of Extracorporeal Photopheresis in Patients with Bronchiolitis Obliterans Syndrome after Lung Transplantation. Transpl Proc (2017) 49:695–8. 10.1016/j.transproceed.2017.02.035 28457374

[32] Del Fante C Scudeller L Oggionni T Viarengo G Cemmi F Morosini M Long-Term Off-Line Extracorporeal Photochemotherapy in Patients with Chronic Lung Allograft Rejection Not Responsive to Conventional Treatment: A 10-Year Single-Centre Analysis. Respiration (2015) 90:118–28. 10.1159/000431382 26112178

[33] Benazzo A Bagnera C Ius F Del Fante C Gottlieb J Hoetzenecker K A European Multi-Center Analysis of Extracorporeal Photopheresis as Therapy for Chronic Lung Allograft Dysfunction. Transpl Int (2024) 36:11551. 10.3389/ti.2023.11551 38282747 PMC10811603

[34] Greer M Liu B Magnusson JM Fuehner T Schmidt BMW Deluca D Assessing Treatment Outcomes in CLAD: The Hannover-Extracorporeal Photopheresis Model. The J Heart Lung Transplant (2023) 42:209–17. 10.1016/j.healun.2022.09.022 37071121

[35] Gautschi F Vogelmann T Ortmanns G Knörr F Steinack C Hage R Early Extracorporeal Photopheresis Treatment Is Associated with Better Survival in Patients with Chronic or Recurrent Acute Lung Allograft Dysfunction. J Clin Apher (2024) 39:e22128. 10.1002/jca.22128 38829041

[36] O’Leary JG Lepe R Davis GL. Indications for Liver Transplantation. Gastroenterology (2008) 134:1764–76. 10.1053/j.gastro.2008.02.028 18471553

[37] Urbani L Mazzoni A Colombatto P Biancofiore G Bindi L Tascini C Potential Applications of Extracorporeal Photopheresis in Liver Transplantation. Transpl Proc (2008) 40:1175–8. 10.1016/j.transproceed.2008.03.071 18555142

[38] Urbani L Mazzoni A De Simone P Catalano G Coletti L Petruccelli S Avoiding Calcineurin Inhibitors in the Early Post‐operative Course in High‐risk Liver Transplant Recipients: The Role of Extracorporeal Photopheresis. J Clin Apher (2007) 22:187–94. 10.1002/jca.20111 17294458

[39] Mazzoni A Giampietro C Bianco I Grazzini T Nencini C Pileggi C Extracorporeal Photopheresis and Liver Transplantation: Our Experience and Preliminary Data. Transfus Apher Sci (2017) 56:515–9. 10.1016/j.transci.2017.07.008 28774829

[40] Xu Z Otrock ZK. Extracorporeal Photopheresis: A Case of Graft-Versus-Host-Disease and Hemophagocytic Lymphohistiocytosis Following Liver Transplantation. Transfusion (Paris) (2022) 62:2409–13. 10.1111/trf.17096 36082758

[41] Houston BL Yan M Tinckam K Kamel-Reid S Chang H Kuo KHM Extracorporeal Photopheresis in Solid Organ Transplant–Associated Acute Graft‐versus‐host Disease. Transfusion (Paris) (2016) 56:962–9. 10.1111/trf.13467 26892365

[42] de Lara Gil M Gil Muñoz M de Lara Gil E Blanquer M Llorente Viñas S Morales Caravaca F Extracorporeal Photopheresis, a Therapeutic Alternative for T Cell-Mediated Rejection in Renal Transplantation. Transpl Proc (2025) 57:16–8. 10.1016/j.transproceed.2024.11.028 39826992

[43] Faenko AP Chuksina YY Zulkarnayev AB Fedulkina VA Vatazin AV Kantaria RO. Immunological Monitoring of the Efficacy of Extracorporeal Photopheresis for Prevention of Kidney Transplant Rejection. Urologiia (2020) 73–8. 10.18565/urology.2020.4.73-78 32897018

[44] Gregorini M Del Fante C Pattonieri EF Avanzini MA Grignano MA Cassaniti I Photopheresis Abates the Anti-HLA Antibody Titer and Renal Failure Progression in Chronic Antibody-Mediated Rejection. Biology (Basel) (2021) 10:547. 10.3390/biology10060547 34207225 PMC8234140

[45] Khush KK Cherikh WS Chambers DC Harhay MO Hayes D Jr Hsich E The International Thoracic Organ Transplant Registry of the International Society for Heart and Lung Transplantation: Thirty-Sixth Adult Heart Transplantation Report — 2019; Focus Theme: Donor and Recipient Size Match. The J Heart Lung Transplant (2019) 38:1056–66. 10.1016/j.healun.2019.08.004 31548031 PMC6816343

[46] Costanzo MR Dipchand A Starling R Anderson A Chan M Desai S The International Society of Heart and Lung Transplantation Guidelines for the Care of Heart Transplant Recipients. The J Heart Lung Transplant (2010) 29:914–56. 10.1016/j.healun.2010.05.034 20643330

[47] Velleca A Shullo MA Dhital K Azeka E Colvin M DePasquale E The International Society for Heart and Lung Transplantation (ISHLT) Guidelines for the Care of Heart Transplant Recipients. The J Heart Lung Transplant (2023) 42:e1–e141. 10.1016/j.healun.2022.10.015 37080658

[48] Ponikowski P Voors AA Anker SD Bueno H Cleland JGF Coats AJS 2016 ESC Guidelines for the Diagnosis and Treatment of Acute and Chronic Heart Failure: The Task Force for the Diagnosis and Treatment of Acute and Chronic Heart Failure of the European Society of Cardiology (ESC)Developed with the Special Contribution of the Heart Failure Association (HFA) of the ESC. Eur Heart J (2016) 37:2129–200. 10.1093/eurheartj/ehw128 27206819

[49] Rubinstein G Valledor AF Moeller CM Baranowska J Oren D Oh DKT Extracorporeal Photopheresis in the Treatment of Cardiac Allograft Rejection in the Modern Era: A Single‐Center Experience. Clin Transpl (2025) 39:e70247. 10.1111/ctr.70247 40698560

[50] Baskaran G Tiriveedhi V Ramachandran S Aloush A Grossman B Hachem R Efficacy of Extracorporeal Photopheresis in Clearance of Antibodies to Donor-Specific and Lung-Specific Antigens in Lung Transplant Recipients. The J Heart Lung Transplant (2014) 33:950–6. 10.1016/j.healun.2014.04.020 24906794 PMC4130746

[51] Vazirani J Routledge D Snell GI Watson D Paraskeva M Westall GP Outcomes Following Extracorporeal Photopheresis for Chronic Lung Allograft Dysfunction Following Lung Transplantation: A Single-Center Experience. Transpl Proc (2021) 53:296–302. 10.1016/j.transproceed.2020.09.003 33039145

[52] Valenzuela NM Reed EF. Antibody-Mediated Rejection across Solid Organ Transplants: Manifestations, Mechanisms, and Therapies. J Clin Invest (2017) 127:2492–504. 10.1172/JCI90597 28604384 PMC5490786

[53] Hidalgo LG Sis B Sellares J Campbell PM Mengel M Einecke G NK Cell Transcripts and NK Cells in Kidney Biopsies From Patients With Donor-specific Antibodies: Evidence for NK Cell Involvement in Antibody-Mediated Rejection. Am J Transplant (2010) 10:1812–22. 10.1111/j.1600-6143.2010.03201.x 20659089

[54] Djamali A Kaufman DB Ellis TM Zhong W Matas A Samaniego M. Diagnosis and Management of Antibody-Mediated Rejection: Current Status and Novel Approaches. Am J Transpl (2014) 14:255–71. 10.1111/ajt.12589 24401076 PMC4285166

[55] Loupy A Toquet C Rouvier P Beuscart T Bories MC Varnous S Late Failing Heart Allografts: Pathology of Cardiac Allograft Vasculopathy and Association with Antibody-Mediated Rejection. Am J Transplant (2016) 16:111–20. 10.1111/ajt.13529 26588356

[56] Gaston RS Cecka JM Kasiske BL Fieberg AM Leduc R Cosio FC Evidence for Antibody-Mediated Injury as a Major Determinant of Late Kidney Allograft Failure. Transplantation (2010) 90:68–74. 10.1097/TP.0b013e3181e065de 20463643

[57] Kueckelhaus M Fischer S Seyda M Bueno EM Aycart MA Alhefzi M Vascularized Composite Allotransplantation: Current Standards and Novel Approaches to Prevent Acute Rejection and Chronic Allograft Deterioration. Transpl Int (2016) 29:655–62. 10.1111/tri.12652 26265179 PMC4785085

[58] Bladon J Taylor PC. Extracorporeal Photopheresis Induces Apoptosis in the Lymphocytes of Cutaneous T‐cell Lymphoma and Graft‐versus‐host Disease Patients. Br J Haematol (1999) 107:707–11. 10.1046/j.1365-2141.1999.01773.x 10606873

[59] Gerber A Bohne M Rasch J Struy H Ansorge S Gollnick H. Investigation of Annexin V Binding to Lymphocytes after Extracorporeal Photoimmunotherapy as an Early Marker of Apoptosis. Dermatology (2000) 201:111–7. 10.1159/000018472 11053912

[60] Cid J Lozano M. Extracorporeal Photopheresis Is Catching up the Pole Position. Bone Marrow Transpl (2021) 56:303–4. 10.1038/s41409-020-01077-w 33024273

[61] Edelson R Wu Y Schneiderman J. American Council on ECP (ACE): Why Now? J Clin Apher (2018) 33:464–8. 10.1002/jca.21627 29575114

[62] Girardi M Berger CL Wilson LD Christensen IR Thompson KR Glusac EJ Transimmunization for Cutaneous T Cell Lymphoma: A Phase I Study. Leuk Lymphoma (2006) 47:1495–503. 10.1080/10428190600581419 16966259

[63] Vowels BR Cassin M Boufal MH Walsh LJ Rook AH. Extracorporeal Photochemotherapy Induces the Production of Tumor Necrosis Factor-α by Monocytes: Implications for the Treatment of Cutaneous T-Cell Lymphoma and Systemic Sclerosis. J Invest Dermatol (1992) 98:686–92. 10.1111/1523-1747.ep12499907 1569319

[64] Gorgun G Miller KB Foss FM. Immunologic Mechanisms of Extracorporeal Photochemotherapy in Chronic Graft-Versus-Host Disease. Blood (2002) 100:941–7. 10.1182/blood-2002-01-0068 12130506

[65] Alcindor T Gorgun G Miller KB Roberts TF Sprague K Schenkein DP Immunomodulatory Effects of Extracorporeal Photochemotherapy in Patients with Extensive Chronic Graft-Versus-Host Disease. Blood (2001) 98:1622–5. 10.1182/blood.v98.5.1622 11520818

[66] Maeda A Schwarz A Bullinger A Morita A Peritt D Schwarz T. Experimental Extracorporeal Photopheresis Inhibits the Sensitization and Effector Phases of Contact Hypersensitivity via Two Mechanisms: Generation of IL-10 and Induction of Regulatory T Cells. The J Immunol (2008) 181:5956–62. 10.4049/jimmunol.181.9.5956 18941184

[67] Biagi E Di Biaso I Leoni V Gaipa G Rossi V Bugarin C Extracorporeal Photochemotherapy Is Accompanied by Increasing Levels of Circulating CD4+CD25+GITR+Foxp3+CD62L+ Functional Regulatory T-Cells in Patients with Graft-Versus-Host Disease. Transplantation (2007) 84:31–9. 10.1097/01.tp.0000267785.52567.9c 17627234

[68] Knobler R Barr ML Couriel DR Ferrara JLM French LE Jaksch P Extracorporeal Photopheresis: Past, Present, and Future. J Am Acad Dermatol (2009) 61:652–65. 10.1016/j.jaad.2009.02.039 19665258

[69] Cho A Jantschitsch C Knobler R. Extracorporeal Photopheresis—An Overview. Front Med (Lausanne) (2018) 5:236. 10.3389/fmed.2018.00236 30211164 PMC6119964

[70] Fisher AJ White M Goudie N Kershaw A Phillipson J Bardgett M Extracorporeal Photopheresis (ECP) in the Treatment of Chronic Lung Allograft Dysfunction (CLAD): A Prospective, Multicentre, Open-Label, Randomised Controlled Trial Studying the Addition of ECP to Standard Care in the Treatment of Bilateral Lung Transplant Patients with CLAD (E-CLAD UK). BMJ Open Respir Res (2024) 11:e001995. 10.1136/bmjresp-2023-001995 38724453 PMC11086459

[71] Arora S Setia R. Extracorporeal Photopheresis: Review of Technical Aspects. Asian J Transfus Sci (2017) 11:81–6. 10.4103/ajts.AJTS_87_16 28970672 PMC5613442

[72] Buder K Zirngibl M Bapistella S Meerpohl JJ Strahm B Bassler D Extracorporeal Photopheresis versus Alternative Treatment for Chronic Graft-Versus-Host Disease after Haematopoietic Stem Cell Transplantation in Children and Adolescents. Cochrane Database Syst Rev (2022) 2022. 10.1002/14651858.CD009898.pub4 35679154 PMC9181448

[73] Saad A de Lima M Anand S Bhatt VR Bookout R Chen G Hematopoietic Cell Transplantation, Version 2.2020, NCCN Clinical Practice Guidelines in Oncology. J Natl Compr Cancer Netw (2020) 18:599–634. 10.6004/jnccn.2020.0021 32519831

[74] Kitko CL Abdel-Azim H Carpenter PA Dalle JH Diaz-de-Heredia C Gaspari S A Prospective, Multicenter Study of Closed-System Extracorporeal Photopheresis for Children with Steroid-Refractory Acute Graft-Versus-Host Disease. Transpl Cell Ther (2022) 28:261.e1–261.e7. 10.1016/j.jtct.2022.01.025 35124293

