# Supplementary material for: Current Usage of Extracorporeal Photopheresis in Solid Organ Transplantations in Europe: A Narrative Review
Source: Transpl Int. 2025 Nov 3;38:14906. doi: 10.3389/ti.2025.14906 (PMC12620306; doi:10.3389/ti.2025.14906)
Supplement: Supplementary file 1 [file DataSheet1.docx]

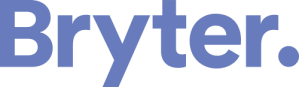

433 Broadway
New York

NY 10013
United States
www.bryter-global.com

| **ECP IN SOLID ORGAN TRANSPLANTATION – QUESTIONNAIRE** |
| --- |

**MODE:** ONLINE SURVEY

**MARKETS** EUROPE

**TARGET TIMING:** 20-25-MINUTES

**TARGET SAMPLE:** TRANSPLANT PHYSICIANS

**KEY TO DOCUMENT FORMATTING:**

- **PURPLE** and **RED TEXT** denotes programming instructions that are NOT shown to respondents
- **SECTION TITLES** are NOT shown to respondents
- **Black text** is used for the main question text respondents will see; ***blue italic text*** denotes respondent instructions

**INTRODUCTION AND SCREENING**

**SHOW TEXT:**

Thank you for your interest in this survey, we appreciate your participation. The European Society of Organ Transplantation (ESOT) is conducting this research aiming to better understand current knowledge and clinical practice for Extra Corporeal Photopheresis in recipients of solid organ transplantation. The survey has been designed by a panel of experts in the field of ECP selected by ESOT. The results of this survey will be included in a scientific peer-reviewed publication.

This research is being conducted and analysed by Bryter, an independent research agency, on behalf of the European Society of Organ Transplantation (ESOT). If you have any questions about the research, you can contact Alisha Hardy at Bryter ([alisha.hardy@bryter-global.com](mailto:alisha.hardy@bryter-global.com)).

Anything you see or read during this research should be treated as confidential. Any information presented during this research is done so solely to explore reactions to such information and should be assumed to represent hypotheses about what can be said about a product or disease area. It should not be used to influence decisions outside the research setting.

This research is being carried out within the codes of conduct of EphMRA and complies with EU / UK data protection law. Any personal information you provide us with will be treated as confidential. You will remain anonymous unless you give permission to be identified. You are free to close this survey at any time. For more information about your rights please see our privacy notice at [http://www.bryter-global.com/privacy-policy](https://www.bryter-global.com/privacy-policy)

We need your consent for us to collect and use any information about you. We won’t keep any personal data you give us for longer than six months after project completion and will only use your personal data for the reasons specified at the time we request it.

At the start of the survey there are a few qualifying questions. The full survey will then take approximately 20-25 minutes to complete.

**AE1 On this basis, do you consent to take part?**

1. Yes
2. No **CLOSE**

**SCREENER**

**SHOW TEXT:**

***These first questions are to ensure we survey an appropriate cross section of eligible physicians and ask relevant questions.***

**SHOW TEXT IF CLOSING AT ANY STAGE IN SCREENER**:

**Thank you for your interest in this survey. Unfortunately, you do not meet the profile of those we are interested in for this particular survey, or we have already completed all the surveys we need with people of a similar profile to you.**

**ASK ALL**

**S1. In which country do you currently practice?**

***Please select one option only.***

SINGLE CODE

1. Austria
2. Belgium
3. Denmark
4. Finland
5. France
6. Germany
7. Greece
8. Ireland
9. Italy
10. Netherlands
11. Norway
12. Poland
13. Portugal
14. Romania
15. Spain
16. Sweden
17. Switzerland
18. Turkey
19. UK
20. Other (SPECIFY) **CLOSE**

**ASK ALL**

**S2. What is your current specialty?**

***Please select one option only.***

SINGLE CODE

1. Transplant Surgeon
2. Cardiac Surgeon
3. Cardiothoracic Surgeon
4. Transplant Cardiologist
5. Transplant Nephrologist
6. Transplant Pulmonologist
7. Transplant Immunologist
8. General Surgeon
9. Nephrologist
10. Hepatologist
11. Haematologist
12. Other (SPECIFY)

**ASK ALL**

**S3. How long have you been practicing as a** <RESPONSE FROM S2>**?**

***Please round to nearest year.***

|  | Years |
| --- | --- |

RECORD NUMBER. RANGE 0-99

**ASK ALL**

**S4.** **For which types of solid organ transplantation are you involved in POST-OPERATIVE treatment and management of patients?**

***Please select all that apply.***

1. Kidney transplantation
2. Heart transplantation
3. Lung transplantation
4. Liver transplantation
5. None of these **CLOSE**

IF S4=1 ONLY – CLASS AS **KIDNEY HCP** AND SHOW **KIDNEY** QUESTIONS AS WELL AS **ASK ALL**

IF S4=2 ONLY – CLASS AS **HEART HCP** AND SHOW **HEART** QUESTIONS AS WELL AS **ASK ALL**

IF S4=3 ONLY – CLASS AS **LUNG HCP** AND SHOW **LUNG** QUESTIONS AS WELL AS **ASK ALL**

IF S4=4 ONLY – CLASS AS **LIVER HCP** AND SHOW **LIVER** QUESTIONS AS WELL AS **ASK ALL**

IF SELECTING MORE THAN ONE CODE, SEE ROUTING INSTRUCTIONS AT S5A-D

**ASK ALL**

**S5a-d. How many** <KIDNEY / HEART / LUNG / LIVER BASED ON PROCEDURES @ S4> **transplants were performed at your centre in 2023?**

***Please provide an estimate if exact numbers cannot be recalled.***

|  | Transplants |
| --- | --- |

RECORD NUMBER.

IF SELECTING MORE THAN ONE CODE AT S4, CLASS AS KIDNEY / HEART / LUNG / LIVER HCP BASED ON THE HIGHEST NUMBER OF TRANSPLANTS PERFORMED @ S5A-D. IF HIGHEST NUMBER @ S5A-D EQUAL, TRY TO GET EQUAL SPLIT OF KIDNEY / HEART / LUNG / LIVER HCPs

**ASK ALL**

**S6.** **Are you involved in the treatment of** <KIDNEY / LUNG / LIVER BASED ON PROCEDURES @ S4 OR S5A-D> **transplant patients for any of the following …?**

***Please select all that apply.***

1. Acute rejection
2. Chronic rejection
3. Recurrent rejection
4. Neither of these **CLOSE**

**ASK ALL**

**S7.** **For which of the following groups of** <KIDNEY / HEART / LUNG / LIVER BASED ON PROCEDURES @ S4 OR S5A-D> **transplant** **patients do you routinely manage post-operative treatment?**

***Please select all that apply.***

1. Adult transplant recipients
2. Paediatric transplant recipients
3. Neither **EXCLUSIVE** **CLOSE**

**SHOW TEXT IF S7= 1 AND 2 (adult and paediatric patients): *Throughout this survey please answer in relation to your ADULT patients only unless otherwise indicated***

**CASELOADS**

**SHOW TEXT: *In this first section, we would like to ask about your post-transplant patient caseloads.***

**ASK ALL**

**Q1a. How many patients Un**

***Please provide an estimate if exact numbers cannot be recalled.***

|  | Patients |
| --- | --- |

RECORD NUMBER. RANGE 0-999

**ASK ALL**

**Q1b. How many of the** <RESPONSE FROM Q1a> < KIDNEY / HEART / LUNG / LIVER BASED ON PROCEDURES @ S4 OR S5A-D> **transplant patients that you currently manage are experiencing rejection / have experienced rejection IN THE LAST 12 MONTHS?**

***Please provide an estimate if exact numbers cannot be recalled.***

|  | Patients |
| --- | --- |

RECORD NUMBER. VALUE CANNOT EXCEED Q1a VALUE

**UNMET NEEDS**

**SHOW TEXT: *Next, we would like to assess the current level of unmet needs for the treatment of*** <KIDNEY / HEART / LUNG BASED ON PROCEDURES @ S4 OR S5A-D> ***transplant rejection.***

**SHOW TEXT IF S7= 1 AND 2 (adult and paediatric patients): *Please continue to answer in relation to your ADULT patients only***

**ASK ALL**

**Q2. What are the greatest unmet needs in terms of the post-transplant management of** <KIDNEY / HEART / LUNG / LIVER BASED ON PROCEDURES @ S4 OR S5A-D> **transplant recipients?**

***Please provide as much detail as possible in the space provided.***

|  |
| --- |

**ASK IF TREAT ACUTE REJECTION (S6=1)**

**Q3a From the following list please rank the top three unmet needs for the management of** <KIDNEY / HEART / LUNG / LIVER BASED ON PROCEDURES @ S4 OR S5A-D> **transplant recipients experiencing T cell mediated (acute cellular) rejection.**

***Please rank up to three of the options below, starting with the most significant unmet need.***

RANDOMISE (ANCHOR OTHER AND NONE OF THESE)

1. Treating / managing symptoms of rejection
2. Lack of treatment efficacy when patients experience rejection
3. Risk of infection
4. Ability to slow disease progression
5. Lack of alternative therapies for rejection / options for later lines of therapy
6. Lack of targeted therapies in case of rejection
7. Lack of personalized treatment for rejection
8. Cost of treatment(s) for rejection
9. Lack of clear diagnosis and definition of disease
10. Risk and subsequent treatment of post-transplant lymphoproliferative disorder (PTLD)
11. [SHOW TO KIDNEY HCPS ONLY] Early calcineurin inhibitor avoidance in patients with pre-existing renal impairment
12. Other (SPECIFY)
13. None of the above – there are no unmet needs **EXCLUSIVE**

**ASK IF TREAT ACUTE REJECTION (S6=1)**

**Q3b From the following list please rank the top three unmet needs for the management of** <KIDNEY / HEART / LUNG / LIVER BASED ON PROCEDURES @ S4 OR S5A-D> **transplant recipients experiencing acute antibody mediated rejection.**

***Please rank up to three of the options below, starting with the most significant unmet need.***

RANDOMISE (ANCHOR OTHER AND NONE OF THESE)

1. Treating / managing symptoms of rejection
2. Lack of treatment efficacy when patients experience rejection
3. Risk of infection
4. Ability to slow disease progression
5. Lack of alternative therapies for rejection / options for later lines of therapy
6. Lack of targeted therapies in case of rejection
7. Lack of personalized treatment for rejection
8. Cost of treatment(s) for rejection
9. Lack of clear diagnosis and definition of disease
10. Risk and subsequent treatment of post-transplant lymphoproliferative disorder (PTLD)
11. [SHOW TO KIDNEY HCPS ONLY] Early calcineurin inhibitor avoidance in patients with pre-existing renal impairment
12. Other (SPECIFY)
13. None of the above – there are no unmet needs **EXCLUSIVE**

**ASK IF TREAT CHRONIC REJECTION (S6=2)**

**Q3c From the following list please rank the top three unmet needs for the management of** <KIDNEY / HEART / LUNG / LIVER BASED ON PROCEDURES @ S4 OR S5A-D> **transplant recipients experiencing chronic rejection.**

***Please rank up to three of the options below, starting with the most significant unmet need.***

RANDOMISE (ANCHOR OTHER AND NONE OF THESE)

1. Treating / managing symptoms of rejection
2. Lack of treatment efficacy when patients experience rejection
3. Risk of infection
4. Ability to slow disease progression
5. Lack of alternative therapies for rejection / options for later lines of therapy
6. Lack of targeted therapies in case of rejection
7. Lack of personalized treatment for rejection
8. Cost of treatment(s) for rejection
9. Lack of clear diagnosis and definition of disease
10. Risk and subsequent treatment of post-transplant lymphoproliferative disorder (PTLD)
11. [SHOW TO KIDNEY HCPS ONLY] Early calcineurin inhibitor avoidance in patients with pre-existing renal impairment
12. Other (SPECIFY)
13. None of the above – there are no unmet needs **EXCLUSIVE**

**PROPHYLACTIC TREATMENT FOR PREVENTION AGAINST ORGAN REJECTION**

**SHOW TEXT: *We would now like to ask you about prophylactic treatment for the prevention of rejection following*** <KIDNEY / HEART / LUNG / LIVER BASED ON PROCEDURES @ S4 OR S5A-D> ***transplant.***

**ASK ALL**

**Q4. What is the clinical rationale for the approach you take regarding treatments to prevent against rejection following** <KIDNEY / HEART / LUNG / LIVER BASED ON PROCEDURES @ S4 OR S5A-D> **transplantation?**

***Please select all that apply***

1. Personal choice
2. Centre-based protocols
3. Clinical trial evidence
4. Registry evidence
5. Other SPECIFY

**TREATMENT OF POST TRANSPLANT REJECTION (ALL REJECTION TYPES)**

**SHOW TEXT: *Next, we would like to ask about the treatment of specific types of post-transplant*** <KIDNEY / HEART / LUNG / LIVER BASED ON PROCEDURES @ S4 OR S5A-D> ***rejection.***

**SHOW TEXT IF S7= 1 AND 2 (adult and paediatric patients): *Once again please consider your ADULT caseloads when answering these questions.***

**ASK ALL**

**Q5a-e** **Overall, how satisfied are you with current treatments for** <T CELL MEDIATED / ACUTE ANTIBODY / CHRONIC ANTIBODY / CHRONIC UNSPECIFIED / RECURRENT> **rejection in** <KIDNEY / HEART / LUNG / LIVER BASED ON PROCEDURES @ S4 OR S5A-D> **transplant** **patients?**

**Please provide an answer using the scale below**

ASK INDICATIONS BASED ON S6 ANSWERS

| S6 CONDITION | INDICATIONS SHOWN |
| --- | --- |
| S6=1 (acute rejection) | 1. T CELL MEDIATED (ACUTE CELLULAR) REJECTION 2. ACUTE ANTIBODY MEDIATED REJECTION |
| S6=2 (chronic rejection) | 1. CHRONIC ANTIBODY MEDIATED REJECTION **DO NOT ASK IF HEART HCP (BASED ON PROCEDURES @ S4 OR S5a-d)** 2. CHRONIC REJECTION – NOT SPECIFIED SHOW AS FOLLOWS BASED ON PROCEDURES @ S4 or S5a-d: KIDNEY HCPs: Chronic rejection – not specified (e.g., chronic allograft nephropathy (CAN)) HEART HCPs: Chronic rejection – not specified (e.g., chronic allograft vasculopathy (CAV)) LUNG HCPs: Chronic rejection – not specified (e.g., chronic lung allograft dysfunction (CLAD)) LIVER HCPs: Chronic rejection – not specified |
| S6=3 (recurrent rejection) | 1. RECURRENT REJECTION |

| Not at all satisfied |  |  |  |  |  | Very satisfied |
| --- | --- | --- | --- | --- | --- | --- |
| 1 | 2 | 3 | 4 | 5 | 6 | 7 |

**ASK ALL**

**Q6a-e** **Overall, how do you rate the performance of treatments for** < T CELL MEDIATED / ACUTE ANTIBODY / CHRONIC ANTIBODY / CHRONIC UNSPECIFIED / RECURRENT> **rejection in** <KIDNEY / HEART / LUNG / LIVER BASED ON PROCEDURES @ S4 OR S5A-D> **transplant patients on the following characteristics?**

***Please use the following scale to evaluate each attribute***

ASK INDICATIONS BASED ON S6 ANSWERS

| S6 CONDITION | INDICATIONS SHOWN |
| --- | --- |
| S6=1 (acute rejection) | 1. T CELL MEDIATED (ACUTE CELLULAR) REJECTION 2. ACUTE ANTIBODY MEDIATED REJECTION |
| S6=2 (chronic rejection) | 1. CHRONIC ANTIBODY MEDIATED REJECTION **DO NOT ASK IF HEART HCP (BASED ON PROCEDURES @ S4 OR S5a-d)** 2. CHRONIC REJECTION – NOT SPECIFIED SHOW AS FOLLOWS BASED ON PROCEDURES @ S4 or S5a-d: KIDNEY HCPs: Chronic rejection – not specified (e.g., chronic allograft nephropathy (CAN)) HEART HCPs: Chronic rejection – not specified (e.g., chronic allograft vasculopathy (CAV)) LUNG HCPs: Chronic rejection – not specified (e.g., chronic lung allograft dysfunction (CLAD)) LIVER HCPs: Chronic rejection – not specified |
| S6=3 (recurrent rejection | 1. RECURRENT REJECTION |

| **1= Terrible** |
| --- |
| **2= Very Poor** |
| **3= Fairly poor** |
| **4= Neither good nor poor** |
| **5= Fairly good** |
| **6= Very Good** |
| **7= Excellent** |
|  |

RANDOMISE ORDER

|  | **All treatments for** T CELL MEDIATED / ACUTE ANTIBODY / CHRONIC ANTIBODY / CHRONIC UNSPECIFIED / RECURRENT **rejection in** KIDNEY / HEART / LUNG / LIVER BASED ON PROCEDURES @ S4 OR S5A-D> **transplant patients** |
| --- | --- |
| 1. Overall efficacy |  |
| 1. Response rate |  |
| 1. Stabilising or reducing the decline in organ function |  |
| 1. Allograft survival |  |
| 1. Mode of administration |  |
| 1. Dosing regimen |  |
| 1. Safety |  |
| 1. Potential to maintain or improve patient quality of life |  |

**ASK KIDNEY HCPs ONLY**

**Q7a-e. Which of the following treatments would you typically prescribe as a first line treatment for** <T CELL MEDIATED / ACUTE ANTIBODY / CHRONIC ANTIBODY / CHRONIC UNSPECIFIED / RECURRENT> **rejection in kidney transplant patients, in addition to any maintenance immunosuppression?**

***Please select all that apply.***

ASK INDICATIONS BASED ON S6 ANSWERS

| S6 CONDITION | INDICATIONS SHOWN |
| --- | --- |
| S6=1 (acute rejection) | 1. T CELL MEDIATED (ACUTE CELLULAR) REJECTION 2. ACUTE ANTIBODY MEDIATED REJECTION |
| S6=2 (chronic rejection) | 1. CHRONIC ANTIBODY MEDIATED REJECTION 2. CHRONIC REJECTION – NOT SPECIFIED Chronic rejection – not specified (e.g., chronic allograft nephropathy (CAN)) |
| S6=3 (recurrent rejection | 1. RECURRENT REJECTION |

1. LIST FOR T CELL MEDIATED
2. Antithymocyte globulin (ATG)
3. Extracorporeal photopheresis (ECP)
4. Oral or IV steroids
5. Active monitoring exclusively (no additional treatment) **EXCLUSIVE**
6. Other (SPECIFY)
7. LIST FOR ACUTE ANTIBODY
8. Alemtuzumab
9. Antithymocyte globulin (ATG)
10. Bortezomib
11. Clazakizumab
12. C1q esterase inhibitors (e.g., Berinert / Cinryze)
13. Eculizumab
14. Extracorporeal photopheresis (ECP)
15. Imlifidase (IdeS; IgG-degrading enzyme of *Streptococcus pyogenes*)
16. Immunoadsorption
17. Intravenous immunoglobulin (IVIG)
18. Plasmapheresis
19. Rituximab
20. Tocilizumab
21. Active monitoring exclusively (no additional treatment) **EXCLUSIVE**
22. Other (SPECIFY)
23. LIST FOR CHRONIC ANTIBODY
24. Alemtuzumab
25. Antithymocyte globulin (ATG)
26. Bortezomib
27. Clazakizumab
28. C1q esterase inhibitors (e.g., Berinert / Cinryze)
29. Eculizumab
30. Extracorporeal photopheresis (ECP)
31. Imlifidase (IdeS; IgG-degrading enzyme of *Streptococcus pyogenes*)
32. Immunoadsorption
33. Intravenous immunoglobulin (IVIG)
34. Plasmapheresis
35. Rituximab
36. Tocilizumab
37. Active monitoring exclusively (no additional treatment) **EXCLUSIVE**
38. Other (SPECIFY)
39. LIST FOR CHRONIC UNSPECIFIED
40. Alemtuzumab
41. Antithymocyte globulin (ATG)
42. Bortezomib
43. Clazakizumab
44. C1q esterase inhibitors (e.g., Berinert / Cinryze)
45. Eculizumab
46. Extracorporeal photopheresis (ECP)
47. Imlifidase (IdeS; IgG-degrading enzyme of *Streptococcus pyogenes*)
48. Immunoadsorption
49. Intravenous immunoglobulin (IVIG)
50. Plasmapheresis
51. Rituximab
52. Tocilizumab
53. Active monitoring exclusively (no additional treatment) **EXCLUSIVE**
54. Other (SPECIFY)
55. LIST FOR RECURRENT
56. Alemtuzumab
57. Antithymocyte globulin (ATG)
58. Bortezomib
59. Clazakizumab
60. C1q esterase inhibitors (e.g., Berinert / Cinryze)
61. Eculizumab
62. Extracorporeal photopheresis (ECP)
63. Imlifidase (IdeS; IgG-degrading enzyme of *Streptococcus pyogenes*)
64. Immunoadsorption
65. Intravenous immunoglobulin (IVIG)
66. Oral or IV steroids
67. Plasmapheresis
68. Rituximab
69. Tocilizumab
70. Active monitoring exclusively (no additional treatment) **EXCLUSIVE**
71. Other (SPECIFY)

**ASK HEART HCPs ONLY**

**Q8a-d. Which of the following treatments would you typically prescribe as a first line treatment for** <T CELL MEDIATED / ACUTE ANTIBODY / RECURRENT> **rejection in heart transplant patients, in addition to any maintenance immunosuppression?**

***Please select all that apply.***

ASK INDICATIONS BASED ON S6 ANSWERS

| S6 CONDITION | INDICATIONS SHOWN |
| --- | --- |
| S6=1 (acute rejection) | 1. T CELL MEDIATED (ACUTE CELLULAR) REJECTION 2. ACUTE ANTIBODY MEDIATED REJECTION |
| S6=2 (chronic rejection) | 1. CHRONIC REJECTION – NOT SPECIFIED Chronic rejection – not specified (e.g., chronic allograft vasculopathy (CAV)) |
| S6=3 (recurrent rejection) | 1. RECURRENT REJECTION |

1. LIST FOR T CELL MEDIATED
2. Antithymocyte globulin (ATG)
3. Extracorporeal photopheresis (ECP)
4. Oral or IV steroids
5. Active monitoring exclusively (no additional treatment) **EXCLUSIVE**
6. Other (SPECIFY)
7. LIST FOR ACUTE ANTIBODY
8. Alemtuzumab
9. Antithymocyte globulin (ATG)
10. Bortezomib
11. Clazakizumab
12. C1q esterase inhibitors (e.g., Berinert / Cinryze)
13. Eculizumab
14. Extracorporeal photopheresis (ECP)
15. Imlifidase (IdeS; IgG-degrading enzyme of *Streptococcus pyogenes*)
16. Immunoadsorption
17. Intravenous immunoglobulin (IVIG)
18. Plasmapheresis
19. Rituximab
20. Tocilizumab
21. Active monitoring exclusively (no additional treatment) **EXCLUSIVE**
22. Other (SPECIFY)
23. LIST FOR CHRONIC UNSPECIFIED
24. Alemtuzumab
25. Antithymocyte globulin (ATG)
26. Bortezomib
27. Antiplatelet agents
28. Eculizumab
29. Extracorporeal photopheresis (ECP)
30. Immunoadsorption
31. Intravenous immunoglobulin (IVIG)
32. Plasmapheresis
33. Rituximab
34. Tocilizumab
35. Statins or other lipid lowering agents
36. Active monitoring exclusively (no additional treatment) **EXCLUSIVE**
37. Other (SPECIFY)
38. LIST FOR RECURRENT
39. Alemtuzumab
40. Antithymocyte globulin (ATG)
41. Bortezomib
42. Clazakizumab
43. C1q esterase inhibitors (e.g., Berinert / Cinryze)
44. Eculizumab
45. Extracorporeal photopheresis (ECP)
46. Imlifidase (IdeS; IgG-degrading enzyme of *Streptococcus pyogenes*)
47. Immunoadsorption
48. Intravenous immunoglobulin (IVIG)
49. Oral or IV steroids
50. Plasmapheresis
51. Rituximab
52. Tocilizumab
53. Active monitoring exclusively (no additional treatment) **EXCLUSIVE**
54. Other (SPECIFY)

**ASK LUNG HCPs ONLY**

**Q9a-e. Which of the following treatments would you typically prescribe as a first line treatment for** <T CELL MEDIATED / ACUTE ANTIBODY / CHRONIC ANTIBODY / CHRONIC UNSPECIFIED / RECURRENT> **rejection in lung transplant patients, in addition to any maintenance immunosuppression?**

***Please select all that apply.***

ASK INDICATIONS BASED ON S6 ANSWERS

| S6 CONDITION | INDICATIONS SHOWN |
| --- | --- |
| S6=1 (acute rejection) | 1. T CELL MEDIATED (ACUTE CELLULAR) REJECTION 2. ACUTE ANTIBODY MEDIATED REJECTION |
| S6=2 (chronic rejection) | 1. CHRONIC ANTIBODY MEDIATED REJECTION 2. CHRONIC REJECTION – NOT SPECIFIED Chronic rejection – not specified (e.g., chronic lung allograft dysfunction (CLAD)) |
| S6=3 (recurrent rejection) | 1. RECURRENT REJECTION |

1. LIST FOR T CELL MEDIATED
2. Antithymocyte globulin (ATG)
3. Extracorporeal photopheresis (ECP)
4. Oral or IV steroids
5. Active monitoring **EXCLUSIVE**
6. Other (SPECIFY)
7. LIST FOR ACUTE ANTIBODY
8. Alemtuzumab
9. Antithymocyte globulin (ATG)
10. Bortezomib
11. Clazakizumab
12. C1q esterase inhibitors (e.g., Berinert / Cinryze)
13. Eculizumab
14. Extracorporeal photopheresis (ECP)
15. Imlifidase (IdeS; IgG-degrading enzyme of *Streptococcus pyogenes*)
16. Immunoadsorption
17. Intravenous immunoglobulin (IVIG)
18. Plasmapheresis
19. Rituximab
20. Tocilizumab
21. Active monitoring exclusively (no additional treatment) **EXCLUSIVE**
22. Other (SPECIFY)

C. LIST FOR CHRONIC ANTIBODY

1. Alemtuzumab
2. Antithymocyte globulin (ATG)
3. Bortezomib
4. Clazakizumab
5. C1q esterase inhibitors (e.g., Berinert / Cinryze)
6. Eculizumab
7. Extracorporeal photopheresis (ECP)
8. Imlifidase (IdeS; IgG-degrading enzyme of *Streptococcus pyogenes*)
9. Immunoadsorption
10. Intravenous immunoglobulin (IVIG)
11. Plasmapheresis
12. Rituximab
13. Tocilizumab
14. Active monitoring exclusively (no additional treatment) **EXCLUSIVE**
15. Other (SPECIFY)

D. LIST FOR CHRONIC UNSPECIFIED

1. Alemtuzumab
2. Antithymocyte globulin (ATG)
3. Bortezomib
4. Clazakizumab
5. C1q esterase inhibitors (e.g., Berinert / Cinryze)
6. Eculizumab
7. Extracorporeal photopheresis (ECP)
8. Imlifidase (IdeS; IgG-degrading enzyme of *Streptococcus pyogenes*)
9. Immunoadsorption
10. Intravenous immunoglobulin (IVIG)
11. Plasmapheresis
12. Rituximab
13. Tocilizumab
14. Active monitoring exclusively (no additional treatment) **EXCLUSIVE**
15. Other (SPECIFY)

E. LIST FOR RECURRENT

1. Alemtuzumab
2. Antithymocyte globulin (ATG)
3. Bortezomib
4. Clazakizumab
5. C1q esterase inhibitors (e.g., Berinert / Cinryze)
6. Eculizumab
7. Extracorporeal photopheresis (ECP)
8. Imlifidase (IdeS; IgG-degrading enzyme of *Streptococcus pyogenes*)
9. Immunoadsorption
10. Intravenous immunoglobulin (IVIG)
11. Oral or IV steroids
12. Plasmapheresis
13. Rituximab
14. Tocilizumab
15. Active monitoring exclusively (no additional treatment) **EXCLUSIVE**
16. Other (SPECIFY)

**ASK LIVER HCPs ONLY**

**Q10a-e. Which of the following treatments would you typically prescribe as a first line treatment for** <T CELL MEDIATED / ACUTE ANTIBODY / CHRONIC ANTIBODY / CHRONIC UNSPECIFIED / RECURRENT> **rejection in liver transplant patients, in addition to any maintenance immunosuppression?**

***Please select all that apply.***

ASK INDICATIONS BASED ON S6 ANSWERS

| S6 CONDITION | INDICATIONS SHOWN |
| --- | --- |
| S6=1 (acute rejection) | 1. T CELL MEDIATED (ACUTE CELLULAR) REJECTION 2. ACUTE ANTIBODY MEDIATED REJECTION |
| S6=2 (chronic rejection) | 1. CHRONIC ANTIBODY MEDIATED REJECTION 2. CHRONIC REJECTION – NOT SPECIFIED |
| S6=3 (recurrent rejection) | 1. RECURRENT REJECTION |

1. LIST FOR T CELL MEDIATED
2. Antithymocyte globulin (ATG)
3. Extracorporeal photopheresis (ECP)
4. Oral or IV steroids
5. Active monitoring exclusively (no additional treatment) **EXCLUSIVE**
6. Other (SPECIFY)
7. LIST FOR ACUTE ANTIBODY
8. Alemtuzumab
9. Antithymocyte globulin (ATG)
10. Bortezomib
11. Clazakizumab
12. C1q esterase inhibitors (e.g., Berinert / Cinryze)
13. Eculizumab
14. Extracorporeal photopheresis (ECP)
15. Imlifidase (IdeS; IgG-degrading enzyme of *Streptococcus pyogenes*)
16. Immunoadsorption
17. Intravenous immunoglobulin (IVIG)
18. Plasmapheresis
19. Rituximab
20. Tocilizumab
21. Active monitoring exclusively (no additional treatment) **EXCLUSIVE**
22. Other (SPECIFY)
23. LIST FOR CHRONIC ANTIBODY
24. Alemtuzumab
25. Antithymocyte globulin (ATG)
26. Bortezomib
27. Clazakizumab
28. C1q esterase inhibitors (e.g., Berinert / Cinryze)
29. Eculizumab
30. Extracorporeal photopheresis (ECP)
31. Imlifidase (IdeS; IgG-degrading enzyme of *Streptococcus pyogenes*)
32. Immunoadsorption
33. Intravenous immunoglobulin (IVIG)
34. Plasmapheresis
35. Rituximab
36. Tocilizumab
37. Active monitoring exclusively (no additional treatment) **EXCLUSIVE**
38. Other (SPECIFY)
39. LIST FOR CHRONIC UNSPECIFIED
40. Alemtuzumab
41. Antithymocyte globulin (ATG)
42. Bortezomib
43. Clazakizumab
44. C1q esterase inhibitors (e.g., Berinert / Cinryze)
45. Eculizumab
46. Extracorporeal photopheresis (ECP)
47. Imlifidase (IdeS; IgG-degrading enzyme of *Streptococcus pyogenes*)
48. Immunoadsorption
49. Intravenous immunoglobulin (IVIG)
50. Plasmapheresis
51. Rituximab
52. Tocilizumab
53. Active monitoring exclusively (no additional treatment) **EXCLUSIVE**
54. Other (SPECIFY)
55. LIST FOR RECURRENT
56. Alemtuzumab
57. Antithymocyte globulin (ATG)
58. Bortezomib
59. Clazakizumab
60. C1q esterase inhibitors (e.g., Berinert / Cinryze)
61. Eculizumab
62. Extracorporeal photopheresis (ECP)
63. Imlifidase (IdeS; IgG-degrading enzyme of *Streptococcus pyogenes*)
64. Immunoadsorption
65. Intravenous immunoglobulin (IVIG)
66. Oral or IV steroids
67. Plasmapheresis
68. Rituximab
69. Tocilizumab
70. Active monitoring exclusively (no additional treatment) **EXCLUSIVE**
71. Other (SPECIFY)

**ASK ALL**

**QNEW. Is there satisfactory supporting evidence for the first line treatment/s you typically prescribe for rejection in** <KIDNEY / HEART / LUNG / LIVER BASED ON PROCEDURES @ S4 OR S5A-D> **transplant patients?**

***Please select one option only.***

1. Yes, for all treatments
2. Yes, but only for some treatments
3. No

**ASK KIDNEY HCPs ONLY**

**Q11a-b. And which of the following treatments would you typically prescribe as a second line treatment for** <ACUTE / CHRONIC > **rejection in kidney transplant patients, in addition to any maintenance immunosuppression?**

***Please select all that apply.***

ASK Q11a IF S6=1 (ACUTE REJECTION)

ASK Q11b IF S6=2 (CHRONIC REJECTION)

1. LIST FOR ACUTE
2. Alemtuzumab
3. Antithymocyte globulin (ATG)
4. Bortezomib
5. Clazakizumab
6. C1q esterase inhibitors (e.g., Berinert / Cinryze)
7. Eculizumab
8. Extracorporeal photopheresis (ECP)
9. Imlifidase (IdeS; IgG-degrading enzyme of *Streptococcus pyogenes*)
10. Immunoadsorption
11. Intravenous immunoglobulin (IVIG)
12. Plasmapheresis
13. Rituximab
14. Tocilizumab
15. Active monitoring exclusively (no additional treatment) **EXCLUSIVE**
16. Other (SPECIFY)
17. LIST FOR CHRONIC
18. Alemtuzumab
19. Antithymocyte globulin (ATG)
20. Bortezomib
21. Clazakizumab
22. C1q esterase inhibitors (e.g., Berinert / Cinryze)
23. Eculizumab
24. Extracorporeal photopheresis (ECP)
25. Imlifidase (IdeS; IgG-degrading enzyme of *Streptococcus pyogenes*)
26. Immunoadsorption
27. Intravenous immunoglobulin (IVIG)
28. Plasmapheresis
29. Re-transplantation
30. Rituximab
31. Tocilizumab
32. Active monitoring exclusively (no additional treatment) **EXCLUSIVE**
33. Other (SPECIFY)

**ASK HEART HCPs IF S6=1 ONLY**

**Q12. And which of the following treatments would you typically prescribe as a second line treatment for acute rejection in heart transplant patients, in addition to any maintenance immunosuppression?**

***Please select all that apply.***

1. Alemtuzumab
2. Antithymocyte globulin (ATG)
3. Bortezomib
4. Clazakizumab
5. C1q esterase inhibitors (e.g., Berinert / Cinryze)
6. Eculizumab
7. Extracorporeal photopheresis (ECP)
8. Imlifidase (IdeS; IgG-degrading enzyme of *Streptococcus pyogenes*)
9. Immunoadsorption
10. Intravenous immunoglobulin (IVIG)
11. Plasmapheresis
12. Rituximab
13. Tocilizumab
14. Active monitoring exclusively (no additional treatment) **EXCLUSIVE**
15. Other (SPECIFY)

**ASK LUNG HCPs ONLY**

**Q13a. And which of the following treatments would you typically prescribe as a second line treatment for** <ACUTE / CHRONIC > **rejection in lung transplant patients, in addition to any maintenance immunosuppression?**

***Please select all that apply.***

ASK Q13a IF S6=1 (ACUTE REJECTION)

ASK Q13b IF S6=2 (CHRONIC REJECTION)

1. LIST FOR ACUTE
2. Alemtuzumab
3. Antithymocyte globulin (ATG)
4. Bortezomib
5. Clazakizumab
6. C1q esterase inhibitors (e.g., Berinert / Cinryze)
7. Eculizumab
8. Extracorporeal photopheresis (ECP)
9. Imlifidase (IdeS; IgG-degrading enzyme of *Streptococcus pyogenes*)
10. Immunoadsorption
11. Intravenous immunoglobulin (IVIG)
12. Plasmapheresis
13. Rituximab
14. Tocilizumab
15. Active monitoring exclusively (no additional treatment) **EXCLUSIVE**
16. Other (SPECIFY)
17. LIST FOR CHRONIC
18. Alemtuzumab
19. Antithymocyte globulin (ATG)
20. Bortezomib
21. Clazakizumab
22. C1q esterase inhibitors (e.g., Berinert / Cinryze)
23. Eculizumab
24. Extracorporeal photopheresis (ECP)
25. Imlifidase (IdeS; IgG-degrading enzyme of *Streptococcus pyogenes*)
26. Immunoadsorption
27. Intravenous immunoglobulin (IVIG)
28. Plasmapheresis
29. Re-transplantation
30. Rituximab
31. Tocilizumab
32. Active monitoring exclusively (no additional treatment) **EXCLUSIVE**
33. Other (SPECIFY)

**ASK LIVER HCPs ONLY**

**Q14a-b. And which of the following treatments would you typically prescribe as a second line treatment for** <ACUTE / CHRONIC> **rejection in liver transplant patients, in addition to any maintenance immunosuppression?**

***Please select all that apply.***

ASK Q14a IF S6=1 (ACUTE REJECTION)

ASK Q14b IF S6=2 (CHRONIC REJECTION)

1. LIST FOR ACUTE
2. Alemtuzumab
3. Antithymocyte globulin (ATG)
4. Bortezomib
5. Clazakizumab
6. C1q esterase inhibitors (e.g., Berinert / Cinryze)
7. Eculizumab
8. Extracorporeal photopheresis (ECP)
9. Imlifidase (IdeS; IgG-degrading enzyme of *Streptococcus pyogenes*)
10. Immunoadsorption
11. Intravenous immunoglobulin (IVIG)
12. Plasmapheresis
13. Rituximab
14. Tocilizumab
15. Active monitoring exclusively (no additional treatment) **EXCLUSIVE**
16. Other (SPECIFY)
17. LIST FOR CHRONIC
18. Alemtuzumab
19. Antithymocyte globulin (ATG)
20. Bortezomib
21. Clazakizumab
22. C1q esterase inhibitors (e.g., Berinert / Cinryze)
23. Eculizumab
24. Extracorporeal photopheresis (ECP)
25. Imlifidase (IdeS; IgG-degrading enzyme of *Streptococcus pyogenes*)
26. Immunoadsorption
27. Intravenous immunoglobulin (IVIG)
28. Plasmapheresis
29. Re-transplantation
30. Rituximab
31. Tocilizumab
32. Active monitoring exclusively (no additional treatment) **EXCLUSIVE**
33. Other (SPECIFY)

**ASK ALL**

**Q15. Is there satisfactory supporting evidence for the second line treatment/s you typically prescribe for** **rejection in** <KIDNEY / HEART / LUNG / LIVER BASED ON PROCEDURES @ S4 OR S5A-D> **transplant patients?**

***Please select one option PER ROW.***

**FOR ALL:**

ONLY SHOW CODES 1 AND 2 IF S6=1 (ACUTE REJECTION)

ONLY SHOW CODES 3 AND 4 IF S6=2 (CHRONIC REJECTION)

ONLY SHOW CODE 5 IF S6=3 (RECURRENT REJECTION)

|  | **Yes, for all treatments** | **Yes, but only for some treatments** | **No** |
| --- | --- | --- | --- |
| 1. T cell mediated (acute cellular) rejection |  |  |  |
| 1. Acute antibody mediated rejection |  |  |  |
| 1. Chronic antibody mediated rejection  **HEART: DO NOT SHOW FOR HEART** |  |  |  |
| 1. Chronic rejection – Not specified  SHOW AS FOLLOWS BASED ON PROCEDURES @ S4 or S5a-d: KIDNEY HCPs: Chronic rejection – not specified (e.g., chronic allograft nephropathy (CAN)) **HEART: DO NOT SHOW FOR HEART** LUNG HCPs: Chronic rejection – not specified (e.g., chronic lung allograft dysfunction (CLAD)) LIVER HCPs: Chronic rejection – not specified |  |  |  |
| 1. Recurrent rejection |  |  |  |

**ASK ALL**

**Q16. Would you say that trying to spare patients from immunosuppression is a key treatment goal?**

***Please select one option only.***

1. Yes
2. No

**ECP AWARENESS AND ACCESS**

**SHOW TEXT: *We would now like to ask you about extracorporeal photopheresis (ECP), specifically.***

**SHOW TEXT IF S7= 1 AND 2 (adult and paediatric patients): *Please consider your ADULT patients only***

**ASK ALL**

**Q17.**  **Are you aware of extracorporeal photopheresis (ECP) as a post-transplant treatment option in the following solid organ transplants?**

***Please select one answer per row***

|  | Aware | Unaware |
| --- | --- | --- |
| 1. Kidney transplantation |  |  |
| 1. Heart transplantation |  |  |
| 1. Lung transplantation |  |  |
| 1. Liver transplantation |  |  |

**ASK ALL**

**Q18. Do you have access to extracorporeal photopheresis (ECP) as a post-transplant treatment option at your centre?**

***Please select one answer***

1. Yes – routinely
2. Yes – but only normally as part of clinical trials
3. No access to ECP at all
4. Don’t know

**ASK IF S7=1 (ADULT TRANSPLANT RECIPIENTS) AND AWARE OF ECP AT Q17 FOR KIDNEY / HEART / LUNG / LIVER BASED ON PROCEDURES @ S4 OR S5A-D AND HAVE ACCESS TO ECP AT Q18 (Q18=1-2)**

**Q19a.** **In the past 12 months, what percentage of your ADULT patients who experienced each of the following types of rejection received treatment with extracorporeal photopheresis (ECP) following their** <KIDNEY / HEART / LUNG / LIVER BASED ON PROCEDURES @ S4 OR S5A-D> **transplant?**

***In each row please type in the % (out of 100% of patients with each type of rejection) receiving ECP. Please provide an estimate if exact percentages cannot be recalled.***

**FOR ALL ADULT-TREATING HCPs:**

ONLY SHOW CODES 1 AND 2 IF S6=1 (ACUTE REJECTION)

ONLY SHOW CODES 3 AND 4 IF S6=2 (CHRONIC REJECTION)

ONLY SHOW CODE 5 IF S6=3 (RECURRENT REJECTION)

|  | % ADULT patients with each type of rejection receiving ECP |
| --- | --- |
| 1. T cell mediated (acute cellular) rejection |  |
| 1. Acute antibody mediated rejection |  |
| 1. Chronic antibody mediated rejection **DO NOT SHOW IF HEART HCP (BASED ON PROCEDURES @ S4 or S5a-d)** |  |
| 1. Chronic rejection – not specified SHOW AS FOLLOWS BASED ON PROCEDURES @ S4 or S5a-d: KIDNEY HCPs: Chronic rejection – not specified (e.g., chronic allograft nephropathy (CAN)) HEART HCPs: Chronic rejection – not specified (e.g., chronic allograft vasculopathy (CAV)) LUNG HCPs: Chronic rejection – not specified (e.g., chronic lung allograft dysfunction (CLAD)) LIVER HCPs: Chronic rejection – not specified |  |
| 1. Recurrent rejection |  |

**ASK IF S7=2 (PAEDIATRIC TRANSPLANT RECIPIENTS) AND AWARE OF ECP AT Q17 FOR KIDNEY / HEART / LUNG / LIVER BASED ON PROCEDURES @ S4 OR S5A-D AND HAVE ACCESS TO ECP AT Q18 (Q18=1-2)**

**Q19b.** **In the past 12 months, what percentage of your PAEDIATRIC patients who experienced each of the following types of rejection received treatment with extracorporeal photopheresis (ECP) following their** <KIDNEY / HEART / LUNG / LIVER BASED ON PROCEDURES @ S4 OR S5A-D> **transplant?**

***In each row please type in the % (out of 100% of patients with each type of rejection) receiving ECP. Please provide an estimate if exact percentages cannot be recalled***

**FOR ALL PAEDIATRIC-TREATING HCPs:**

ONLY SHOW CODES 1 AND 2 IF S6=1 (ACUTE REJECTION)

ONLY SHOW CODES 3 AND 4 IF S6=2 (CHRONIC REJECTION)

ONLY SHOW CODE 5 IF S6=3 (RECURRENT REJECTION)

|  | % PAEDIATRIC patients with each type of rejection receiving ECP |
| --- | --- |
| 1. T cell mediated (acute cellular) rejection |  |
| 1. Acute antibody mediated rejection |  |
| 1. Chronic antibody mediated rejection **DO NOT SHOW IF HEART HCP (BASED ON PROCEDURES @ S4 or S5a-d)** |  |
| 1. Chronic rejection – not specified SHOW AS FOLLOWS BASED ON PROCEDURES @ S4 or S5a-d: KIDNEY HCPs: Chronic rejection – not specified (e.g., chronic allograft nephropathy (CAN)) HEART HCPs: Chronic rejection – not specified (e.g., chronic allograft vasculopathy (CAV)) LUNG HCPs: Chronic rejection – not specified (e.g., chronic lung allograft dysfunction (CLAD)) LIVER HCPs: Chronic rejection – not specified |  |
| 1. Recurrent rejection |  |

**SHOW TEXT IF S7= 1 AND 2 (adult and paediatric patients): *Please now consider your ADULT patients only for the remainder of this survey.***

**ASK IF ANY AT Q19a OR 19b > 0%**

**Q20. How would you specifically characterize the patients who receive extracorporeal photopheresis (ECP) for the management of their** <KIDNEY / HEART / LUNG / LIVER BASED ON PROCEDURES @ S4 OR S5A-D> **transplant?**

***Please describe in your answer what makes them stand out among all*** <KIDNEY / HEART / LUNG / LIVER BASED ON PROCEDURES @ S4 OR S5A-D> ***transplant patients. Please do not include any personal data.***

|  |
| --- |

**ASK ALL**

**Q21.** **Below are several factors that may or may not be barriers to wider use of extracorporeal photopheresis (ECP) in the management of** <KIDNEY / HEART / LUNG / LIVER BASED ON PROCEDURES @ S4 OR S5A-D> **transplant recipients.**

**Please rank the top three factors that you think are currently limiting wider ECP use, starting with the most significant barrier**

1. Reimbursement challenges / cost
2. Guidelines restrict usage
3. Lack of standardised protocols
4. Safety concerns
5. It is not the standard of care
6. Satisfaction with alternative treatment(s)
7. Lack of personal experience
8. Patient factors (poor venous access, anticoagulation, low haematocrit)
9. Lack of clinician understanding of ECP’s mechanism of action
10. Other (SPECIFY)
11. None of these **EXCLUSIVE**

**ASK ALL**

**Q22.** **To what extent do you agree or disagree with the following statements regarding extracorporeal photopheresis (ECP) for** <KIDNEY / HEART / LUNG / LIVER BASED ON PROCEDURES @ S4 OR S5A-D> **transplant recipients?**

***Please use the following scale to indicate agreement / disagreement with each statement .***

RANDOMISE

1. ECP is an effective prophylactic treatment against and / or curative treatment of <KIDNEY / HEART / LUNG / LIVER BASED ON PROCEDURES @ S4 OR S5A-D> T cell mediated (acute cellular) transplant rejection
2. ECP is an effective prophylactic treatment against and / or curative treatment of <KIDNEY / HEART / LUNG / LIVER BASED ON PROCEDURES @ S4 OR S5A-D> acute antibody mediated transplant rejection
3. DO NOT ASK HEART HCPs: ECP is an effective prophylactic treatment against and / or curative treatment of <KIDNEY / LIVER / LUNG BASED ON PROCEDURES @ S4 OR S5A-D> chronic antibody mediated transplant rejection
4. ECP is more effective for prophylactic treatment of transplant rejection as opposed to curative treatment of transplant rejection
5. ECP is typically reserved for <KIDNEY / HEART / LUNG / LIVER BASED ON PROCEDURES @ S4 OR S5A-D> transplant rejection patients where standard therapies have failed
6. There is a lack of strong clinical trial evidence demonstrating efficacy of ECP in <KIDNEY / HEART / LUNG / LIVER BASED ON PROCEDURES @ S4 OR S5A-D> transplant rejection
7. There is a lack of strong clinical trial evidence supporting safety of ECP in ALL TYPES of solid organ transplant rejection
8. It would be of benefit to my clinical practice if more ECP was used to <KIDNEY / HEART / LUNG / LIVER BASED ON PROCEDURES @ S4 OR S5A-D> transplant rejection in the future

| Strongly disagree  1 | 2 | 3 | 4 | 5 | 6 | Strongly agree  7 | Don’t know  99 |
| --- | --- | --- | --- | --- | --- | --- | --- |

**Q23. What is the most significant thing that you think would encourage greater use of extracorporeal photopheresis (ECP) in the management of** <KIDNEY / HEART / LUNG / LIVER BASED ON PROCEDURES @ S4 OR S5A-D> **transplant recipients?**

***Please provide as much detail as possible in the space provided.***

|  |
| --- |

**Q24. Which of the following would encourage greater use of extracorporeal photopheresis in the management of** <KIDNEY / HEART / LUNG / LIVER BASED ON PROCEDURES @ S4 OR S5A-D> **transplant recipients in the future?**

***Please select all that apply***

RANDOMISE (ANCHOR OTHER AND NONE)

1. More robust trial data in solid organ transplant for the prevention of rejection
2. More robust trial data in solid organ transplant for the treatment of rejection
3. More robust trial data in solid organ transplant demonstrating safety
4. Education on how to select candidates for ECP
5. Education on ECP mechanism of action / benefits
6. Stronger cost-benefit data
7. Development of a standardised protocol / technology for ECP that would feature in local or national guidelines
8. Research into biomarkers that could predict ECP effectiveness in solid organ transplantation
9. Other SPECIFY OE BOX
10. None of the above EXCLUSIVE

**SURVEY END**

**THANK & CLOSE**

Thank you for taking part in this study.

Should you have any queries on how your data is used, you can contact our Data Protection Officer via email: [dpo@bryter-uk.com](mailto:dpo@bryter-uk.com). Our privacy statement is available here: <https://www.bryter-global.com/privacy-policy>
